# Genetic Analysis of T Cell Lymphomas in Carbon Ion-Irradiated Mice Reveals Frequent Interstitial Chromosome Deletions: Implications for Second Cancer Induction in Normal Tissues during Carbon Ion Radiotherapy

**DOI:** 10.1371/journal.pone.0130666

**Published:** 2015-06-30

**Authors:** Benjamin J. Blyth, Shizuko Kakinuma, Masaaki Sunaoshi, Yoshiko Amasaki, Shinobu Hirano-Sakairi, Kanae Ogawa, Ayana Shirakami, Yi Shang, Chizuru Tsuruoka, Mayumi Nishimura, Yoshiya Shimada

**Affiliations:** Radiobiology for Children’s Health Program, Research Center for Radiation Protection, National Institute of Radiological Sciences, Chiba, Japan; ENEA, ITALY

## Abstract

Monitoring mice exposed to carbon ion radiotherapy provides an indirect method to evaluate the potential for second cancer induction in normal tissues outside the radiotherapy target volume, since such estimates are not yet possible from historical patient data. Here, male and female B6C3F1 mice were given single or fractionated whole-body exposure(s) to a monoenergetic carbon ion radiotherapy beam at the Heavy Ion Medical Accelerator in Chiba, Japan, matching the radiation quality delivered to the normal tissue ahead of the tumour volume (average linear energy transfer = 13 keV.μm^-1^) during patient radiotherapy protocols. The mice were monitored for the remainder of their lifespan, and a large number of T cell lymphomas that arose in these mice were analysed alongside those arising following an equivalent dose of ^137^Cs gamma ray-irradiation. Using genome-wide DNA copy number analysis to identify genomic loci involved in radiation-induced lymphomagenesis and subsequent detailed analysis of *Notch1*, *Ikzf1*, *Pten*, *Trp53* and *Bcl11b* genes, we compared the genetic profile of the carbon ion- and gamma ray-induced tumours. The canonical set of genes previously associated with radiation-induced T cell lymphoma was identified in both radiation groups. While the pattern of disruption of the various pathways was somewhat different between the radiation types, most notably *Pten* mutation frequency and loss of heterozygosity flanking *Bcl11b*, the most striking finding was the observation of large interstitial deletions at various sites across the genome in carbon ion-induced tumours, which were only seen infrequently in the gamma ray-induced tumours analysed. If such large interstitial chromosomal deletions are a characteristic lesion of carbon ion irradiation, even when using the low linear energy transfer radiation to which normal tissues are exposed in radiotherapy patients, understanding the dose-response and tissue specificity of such DNA damage could prove key to assessing second cancer risk in carbon ion radiotherapy patients.

## Introduction

One of the rationales for the use of heavy ion radiotherapy in cancer treatment is the ability to minimise the adverse effects in normal tissue by minimizing the radiation dose received outside the target volume [1, 2]. The reduction in dose can be measured directly, but assessing the reduction in adverse normal tissue effects is complicated by the uncertainty of the relative biological effectiveness of the heavy ions compared to standard photon radiotherapy [3]. For deterministic adverse events (tissue reactions which show dose-thresholds), the success of heavy ion radiotherapy protocols can be assessed directly in patients; however, the risk of stochastic effects (specifically, induction of second cancers) after heavy ion radiotherapy is more difficult to assess due to the challenges of conducting an appropriately powered epidemiology study, given the inherent signal-to-noise issues associated with radiation-induced cancer [4].

Although the accelerated heavy particles in carbon ion radiotherapy are ranged to stop within the target volume delivering their energy with high linear energy transfer (LET)–which underlies the increased tumour control per unit dose–the same particles first pass through normal (non-target) tissues ahead of the target (reviewed in [5]). During their transit through the normal tissue the particles’ greater initial energy results in fewer interactions per unit distance, and consequently, they have a lower LET than when depositing energy within the target volume. It is thus exposure of normal tissue to the lower LET component of the carbon ion radiation LET-depth curve which poses the risk for induction of second cancers following high LET carbon radiotherapy [6].

Early research in mice showed equivocal differences in cancer incidence for exposure to a relatively low dose of 0.426 Gy of high LET carbon ions within the spread-out Bragg peak (SOBP) compared to a similar dose of X-rays [7]. A study in radiation-induced mammary carcinoma-sensitive female rats using a larger dose-range of similar SOBP carbon ions clearly showed more efficient mammary carcinoma induction per unit dose than gamma ray-irradiations [8], yet exposure to carbon ions at lower LET gave relative biological effectiveness values for mammary carcinoma induction ranging from 0.2 up to 3.3 [9] with a strong modification by age-at-exposure, a key parameter in mammary tumourigenesis. Given that children exposed to radiation are generally at greater relative cancer risk than similarly exposed adults [10–12] (though this is not a universal finding [13, 14]) the induction of second cancers in children is of particular concern when considering the safety of carbon ion radiotherapy relative to traditional photon radiotherapy.

Cancers arising from precursor or immature T cells developing within the thymus [15], presenting as massive clonal expansion of thymic T cells (with or without involvement of peripheral lymphoid organs), are one of the principal tumours induced in mice exposed to whole-body irradiation (reviewed in [16]). Although the incidence following irradiation varies across inbred mouse strains, T cell lymphomas (TL) generally have short latency (within 3–9 months following irradiation), occur infrequently in control mice, and show characteristic changes in one or more key tumour suppressor genes (TSG) or oncogenes [17–20]. Although there are several key differences [21], the chromosomal and genetic changes in mouse TL largely overlap with those seen in human T cell acute lymphoblastic leukaemia (T-ALL) [22–24]. Almost all of the well-defined human leukaemogens are leukaemogenic in mice [25]; although the leukaemic subtypes may differ, even when the mechanism of action can be demonstrated to be the same at the cellular/molecular level [25]. As such, TL provide a useful endpoint for directly comparing cancer risks while varying radiation dose, dose-rate, fractionation schedule, or radiation quality *in vivo*. Currently, no information has been reported on the genetic characteristics of carbon ion-induced tumours from *in vivo* experiments, and studies in normal human cells from radiotherapy patients are only preliminary [26].

In this study, a large number of TL induced by carbon ion irradiation (using the lower LET range relevant to normal tissue exposure in human patients) were analysed in detail to characterise the pattern of genetic changes. Tumours were induced by 4–4.8 Gy radiation doses, with one experiment delivering the dose in a single exposure, the other delivering the dose over four fractions, each one week apart. We aimed to determine whether the mechanisms underlying TL induced by low LET carbon ion irradiation were common to or distinct from those observed in TL induced by gamma ray irradiation, and found that although the canonical set of radiation-induced TL genes were involved with either irradiation type, the frequency of large interstitial deletions was higher following carbon ion irradiation.

## Materials and Methods

### Mice and Irradiations

Male and female F1 hybrid mice from timed-mating of C57BL/6NCrlCrlj female and C3H/HeNCrlCrlj male breeders sourced from Charles River Japan were born into our specific pathogen free (SPF) animal housing facility at the National Institute of Radiological Sciences (Chiba, Japan). Routine screening of monitor mice confirmed maintenance of SPF conditions. The mice used in the present study were all irradiated as part of three independent large-scale experiments conducted between 2006 and 2013. Three to four days after birth, mice were sexed and randomly re-distributed between the nursing mothers to balance litter sizes and sex (with at least 40 males and 40 females per irradiation group). All of the infant mice from within each new randomized litter were assigned to the same treatment group. Mice were joint-housed with up to five mice per aluminium cage after weaning at 28 days old, with the cage changed weekly. Mice were provided with wood shaving bedding and fed sterilised stock ration and water *ad libitum* (provided fresh twice weekly), and maintained at 22°C under a 12-h light-dark cycle. Mice in the first experiment were exposed to a single dose of 0–4.0 Gy gamma-rays using a ^137^Cs source (Gammacell-40, Nordion) at 1 week of age, at a dose-rate of 0.5 Gy.min^-1^. This exposure was performed inside the SPF animal housing facility using a ventilated acrylate polymer enclosure.

Mice in the second experiment were transferred (approximately 200 m distance) to the Heavy Ion Medical Accelerator in Chiba (HIMAC, Chiba, Japan) building at 1 week of age in air-tight acrylate polymer enclosures (each supplied with 0.22 μm PVDF filter-sterilized air) in which they remained during their radiation exposure. The mice were exposed to a total carbon radiation dose of 0–4.8 Gy of carbon ions in a single exposure and were then delivered back to their SPF housing facility, and returned to their standard housing. Mice in the third experiment were similarly transferred to the HIMAC building starting at 1 week of age, where they ultimately received 4 doses (1 dose per week) of 0–1.2 Gy, also for a total radiation dose of 0–4.8 Gy. These mice were returned to their standard housing in the SPF housing facility between each fraction and after receiving their final dose. In both experiments, the irradiations were conducted with a monoenergetic beam (290 MeV.u^-1^) of accelerated ^12^C ions at a nominal dose-rate of 4.0 Gy.min^-1^ (for the 4.0 and 4.8 Gy single exposures) or 1.0 Gy.min^-1^ (for the 1.0 and 1.2 Gy fractions) by the addition of a mesh-attenuator which reduced the particle fluence without significantly altering the beam characteristics. Taking into account the size of the mice and the shielding provided by the plastic enclosure, the whole body of each mouse was traversed by carbon ions with a dose-averaged LET estimated at 13 keV.μm^-1^. Dosimetry for each mouse was automatically controlled by the exposure system, which was independently verified by technical staff before each exposure session.

Irradiated mice were monitored daily until they were sacrificed shortly before their natural death, and were considered moribund when they exhibited the hallmark signs of TL (including shallow and rapid breathing, pallor, hunched posture, ruffled coat or lethargic movement). Mice were sacrificed by exsanguination under terminal anaesthesia with inhaled isofluorane in air, before detailed necropsy and tissue-banking. All procedures involving animals were approved by the Institutional Animal Care and Use Committee of the National Institute of Radiological Sciences (Inage, Chiba, Japan) (Approval Numbers 07–1017 and 10–2019) and all efforts were undertaken to minimize suffering.

### Cohort Selection and Sample Processing

From the three experiments described above, five treatment groups were selected from a database containing the experimental and necropsy details, namely: 1 × 4.0 Gy (n = 82), 1 × 4.8 Gy (n = 88), 4 × 1.0 Gy (n = 80) and 4 × 1.2 Gy (n = 80) carbon ion irradiation, and 1 × 4.0 Gy gamma ray irradiation (n = 98). Each experimental group contained at least 40 males and 40 females, and all mice that met the following criteria (between 15 and 38 per group) were selected for inclusion in the genetic analysis study (S1 Table): mice were sacrificed when moribund and provisionally diagnosed at necropsy with TL (with or without extra-thymic tumour involvement) with a thymus mass greater than 150 mg (twice the average peak thymus weight in unirradiated mice of this strain). The remaining experimental groups using lower radiation doses were not included due to the small number of tumours available per group (only 15 qualifying TL from a total of 950 mice in the remaining dose groups). Extraction of DNA and RNA, and preparation of cDNA are detailed in the supporting information. For some mice, cell suspensions from the enlarged thymus were also processed immediately for immunophenotyping by flow cytometry using a panel of fluorescent-conjugated primary antibodies against the cell surface antigens CD4, CD8, CD90 and CD45R.

### DNA Copy Number Variation Analysis

DNA copy number variation across the tumour genome was assessed for 12 tumours arising in the gamma ray irradiation group and 20 tumours in mice irradiated with carbon ions (4–6 per carbon treatment group) using array-based comparative genomic hybridisation (array CGH). The data discussed in this publication have been deposited in NCBI's Gene Expression Omnibus [27] and are accessible through GEO Series accession number GSE61315 (http://www.ncbi.nlm.nih.gov/geo/query/acc.cgi?acc=GSE61315). Isolated tumour and reference (sex-matched ear) DNA was treated with RNase before re-extraction with phenol-chloroform, and clean-up by ethanol precipitation. DNA mass was re-quantified before restriction enzyme digestion, fluorescent labelling of fragments using the *SureTag Complete DNA labelling kit* (Agilent) and hybridisation to customised 8 × 60k CGH arrays (GPL19183) following the manufacturer’s protocol. The arrays consisted of probe-sets tiled across the whole genome (mean of 1 probe per 74 kb), medium density coverage (50 probes per gene) of 24 TSG and oncogenes known to be involved in human and mouse T cell leukaemia/lymphoma [22–24], and high density coverage (1 probe per 100–200 bp) of a further 15 primary candidate loci including *Trp53* (MGI: 98834), *Pten* (MGI: 109583), *Ikzf1* (MGI: 1342540), *Bcl11b* (MGI: 1929913), *Cdkn2a* (MGI: 104738) and *Notch1* (MGI: 97363). The array slides were processed and scanned following the manufacturer’s instructions. Copy number variations were identified using the *Genomic Workbench Lite Edition 7*.*0* (Agilent) using probe-quality weighted interval scores (ADM-2, Threshold = 5.0) after exclusion of probes with saturated signals and default normalisation and centralisation of the data. DNA copy number was assessed at and adjacent to the *Bcl11b* locus across the whole tumour cohort by real-time quantitative PCR relative to a *Tert* (MGI: 1202709) 2-copy reference target.

### Loss of Heterozygosity Analysis

Loss of heterozygosity (LOH) was assessed at or flanking the genes *Ikzf1*, *Bcl11b* and *Pten*, and for those tumours analysed by array CGH, also at locations along the length of chromosomes 11, 12 and 19, respectively. DNA was amplified by PCR with primers flanking sites known to be polymorphic for sequence length between the parental strains. LOH was determined by the loss of either of the amplicons representing the parental alleles, detected either by melt curve analysis using a fluorescent DNA intercalating dye, high-resolution agarose gel electrophoresis or capillary gel electrophoresis (depending on the increasing resolution required to differentiate the parental allele amplicons).

### Sequence Analysis

Protein coding regions were amplified by PCR from tumour cDNA using primers to produce a single amplicon from the *Pten* and *Trp53* genes and several overlapping fragments from the *Ikzf1* gene. For *Notch1*, primers flanking the HD domain and flanking the PEST domain were used to amplify these two regions known to harbour the majority of *Notch1* activating mutations [28]. PCR amplicons were isolated by excising bands after agarose gel electrophoresis, which were then purified and subjected to direct DNA sequencing using the original PCR primers and in some cases, additional internal primers. For novel *Ikzf1* isoforms produced due to mutation-induced exon skipping, real-time RT-PCR across exon-exon junctions was also used to prevent false-negatives from failed amplification due to loss of primer binding sites. To validate frameshift mutations occurring at high frequency by insertion/deletion mutations at several homopolymer tracts in the cDNA, *Ikzf1* exons 4 and 7, and *Pten* exons 5, 6 and 8 were also sequenced from the DNA of relevant tumours to demonstrate they were *bona fide* mutations and not errors introduced during reverse transcription or PCR amplification.

### Analysis of Recombination-Mediated Deletions

The presence of a specific recombination-mediated deletion in the 5′ region of *Notch1* was assessed by PCR using primers flanking the presumptive upstream and downstream breakpoints [28]. Tumours positive for this specific deletion were identified by the presence of the amplicon by agarose gel electrophoresis. In a number of cases, the amplicons were excised and sequenced using both PCR primers which confirmed the presence of the specific breakpoint.

### Statistics and Analysis Software

DNA sequencing traces were visualised and alignments prepared using the software ATGC v7.5.1 (Genetyx Corporation) and mutations were identified manually by divergence from the reference sequences (S1 Methods). Cohort survival data was analysed and graphed using the freely available software KMWin v1.51 [29]. Survival curves were compared by pairwise log-rank tests. Remaining statistical analyses were performed using IBM SPSS Statistics v. 22 (IBM Corporation). Associations in contingency tables were tested using either Fisher’s exact test or Pearson’s Chi-squared test. Differences in the distribution of ordinal variables were tested by the Mann-Whitney U test. Where applicable, all tests were two-tailed, and as per convention, *P* values were considered significant when *P* < 0.05.

## Results

### Tumour Incidence and Survival

**Fig 1:**
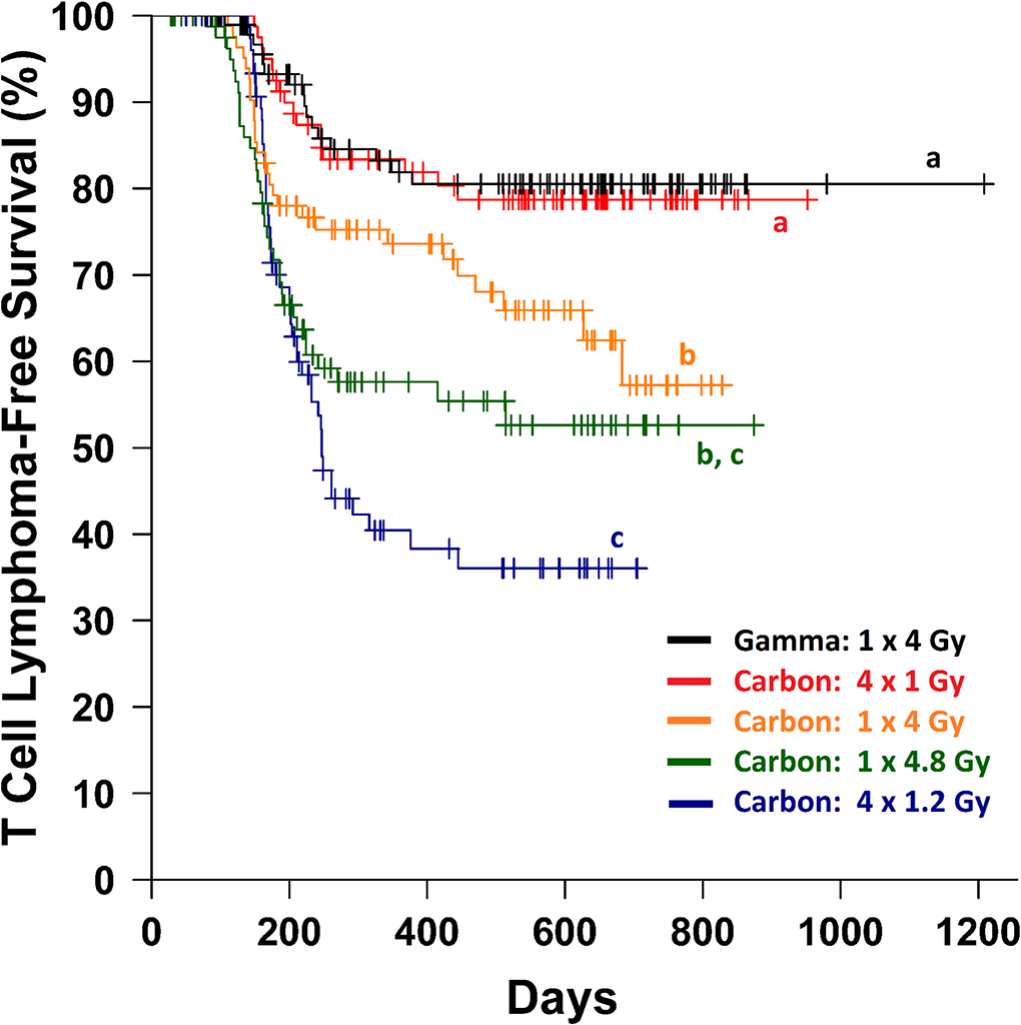
T Cell Lymphoma-Free Survival of Mice Irradiated with Gamma Rays or Carbon Ions. The Kaplan-Meier estimator is plotted for the five irradiation groups with censored cases (causes of death other than TL) marked with crosses. Curves sharing the same letter designation (a, b or c) are not significantly different by pairwise log-rank tests (*P* > 0.05).

Mice irradiated with a single 4 Gy dose of carbon ions showed a higher crude TL incidence (*P* = 0.014, Fisher’s Exact, S1 Table) and shorter TL-free survival (*P* = 0.01, Log-Rank, Fig 1) than their counterparts exposed to a single 4 Gy dose of gamma rays. The use of four fractions each separated by one week models the exposure of normal tissue to a clinical regimen of four doses a week for four weeks, where the beam-entry direction is rotated by 90° each day (sparing each field of normal tissue until the following week). When the lower carbon ion dose of 4 Gy was fractionated, TL-free survival was prolonged to mirror that of the gamma ray acute exposure group with a decreased TL incidence compared to the 4 Gy single carbon ion exposure. Surprisingly, when the 4.8 Gy carbon ion dose was fractionated the TL incidence increased (*P* = 0.06, Fisher’s Exact), but without significantly reducing TL-free survival (*P* = 0.24, Log-Rank) given the competing risk from non-TL deaths as the two survival curves diverged. Apart from TL, remaining causes of death in the first year were mostly leukaemia/lymphomas involving the spleen and/or lymph nodes (and not the thymus), while eventually solid tumours dominated by those involving the liver and lungs were responsible for deaths between 1 and 2 years of age. Acute deaths (<100 days old, non-cancer) were restricted to the 4.8 Gy carbon exposure groups (*n* = 9, single fraction; *n* = 4, four fractions) plus four acute deaths following 4 Gy gamma ray exposure.

Thirty-five sequential thymus-associated lymphomas from carbon ion irradiated mice were analysed by flow cytometry for their expression of B and T lymphocyte associated cell-surface markers (CD45R and CD90, respectively) as well as CD4 and CD8 expression, which distinguish the differentiation stages of maturing T cells. All of the tumours were CD90-positive and CD45R-negative consistent with a diagnosis of TL, with the full range of immunophenotypes represented including CD4/CD8 double positive, double negative and single positive tumours, tumours where CD4 and/or CD8 were expressed along a continuum from high to low, and tumours with multiple distinct immunophenotypes (S3 Table).

### Genome-Wide DNA Copy Number Analysis

In order to assess the involvement of known targets in radiation-induced mouse TL (and/or human T-ALL) in TL from carbon ion-irradiated mice, and to uncover any novel sites of deletion or amplification, a sub-group of twenty TL arising in mice from the four carbon ion-irradiated groups (C-TL), and twelve TL arising in the gamma ray-irradiated mice (G-TL) were chosen for genome-wide DNA copy number analysis by array CGH (S4 Table). Putative trisomy 15 was present within 21 of the 32 tumours (with amplification of most of chromosome 15 in a further two TL), while putative trisomy 14 was observed in 11 of 32 tumours (Table 1) with no evidence of association between the two events or with either radiation type (Table 2). We have previously reported the occurrence of trisomy 14 in X ray-induced mouse TL with similar frequency to that observed here [18]; however unlike the well documented incidence of trisomy 15, to our knowledge these are the first reports of recurrent gains of chromosome 14. Each tumour showed copy number variation at immunoglobulin heavy chain and/or T cell receptor loci indicative of gene rearrangement at these sites (S1 Fig). In addition, specific rearrangement at the Skint immunoglobulin superfamily (MGI:3649262) region of chromosome 4 [30] was identified in 9 of 32 tumours. Excluding these lymphocyte development-associated somatic rearrangement sites, the most common specific sites of copy number loss were on chromosomes 2, 4, 11, 12 and 19; either within, or including *Notch1*, *Cdkn2a*, *Ikzf1*, *Bcl11b* and *Pten*, respectively.

**Table 1:** Summary of DNA copy number aberrations identified in twenty C-TL and twelve G-TL.

Chromosome	Locus	Aberration Class	C-TL Frequency (*n*/20)	G-TL Frequency (*n*/12)
1		Centromeric Amplification	1	0
1		Interstitial Amplification	0	1
1		Telomeric Amplification	3	1
1		Trisomy	1	0
1	Including *Bend6*	Large Interstitial Deletion	1	0
1	Including *Abl2*	Large Interstitial Deletion	1	0
2		Telomeric Amplification	1	0
2	Including *Abi1*	Large Interstitial Deletion	1	0
2	*Notch1* (5ʹ Deletion)	Recurrent Small Deletion	7	5
2	Inside *Notch1*	Small Interstitial Deletion	1	0
3		Centromeric Deletion	2	0
3		Telomeric Deletion	0	1
3	Including *Vcam1*	Large Interstitial Deletion	1	0
4	Breakpoint at *Tal2*	Centromeric Amplification	0	1
4	Breakpoint at *Cdkn2a*	Interstitial Amplification	1	0
4	Inside or including *Cdkn2a*	Small Interstitial Deletion	5	2
4	Including *Tox*	Large Interstitial Deletion	0	1
4	Compound Deletions in *Pax5*	Large Interstitial Deletion	0	1
5		Telomeric Amplification	1	1
5		Telomeric Deletion	1	0
5	Including *Rad9b*	Large Interstitial Deletion	0	1
6		Interstitial Amplification	1	0
6		Trisomy	0	1
6	Breakpoint at *Wnt2*	Large Interstitial Deletion	1	0
6		Large Interstitial Deletion	1	0
7		Interstitial Amplification	1	0
7		Large Interstitial Deletion	1	0
8		Telomeric Amplification	0	1
8		Large Interstitial Deletion	1	0
9		Interstitial Amplification	1	0
9	Including *Suhw4*	Large Interstitial Deletion	1	0
9	Breakpoint at *Tgfbr2*	Large Interstitial Deletion	1	0
10		Interstitial Amplification	1	0
10		Telomeric Amplification	0	1
10		Trisomy	2	2
11		Centromeric Amplification	0	1
11		Centromeric Deletion	2	1
11		Telomeric Amplification	2	1
11	Inside or including *Ikzf1*	Small Interstitial Deletion	5	6
11	Including *Ikzf1*	Large Interstitial Deletion	4	0
11	Inside *Ikzf1*	Interstitial Amplification	1	0
12		Centromeric Amplification	4	1
12	Breakpoint at *Sox11*	Centromeric Deletion	1	0
12	Upstream of *Sox11*	Large Interstitial Deletion	1	0
12	Including or Breakpoint at *Bcl11b*	Telomeric Deletion	10	4
12	Inside or including *Bcl11b*	Large Interstitial Deletion	5	1
14	Breakpoint at *TCRa/d*	Centromeric Deletion	1	1
14		Trisomy	8	3
15		Centromeric Amplification	1	1
15		Trisomy	13	8
16		Centromeric Deletion	3	0
16		Telomeric Amplification	1	2
16		Trisomy	0	1
16	Upstream of *A2bp1*	Large Interstitial Deletion	1	0
17		Telomeric Deletion	1	0
17		Trisomy	1	0
17	Breakpoint at *Cdkn1a*	Large Interstitial Deletion	0	1
18		Centromeric Amplification	1	0
19	Compound Deletions in *Pten*	Small Interstitial Deletion	4	0
19	Inside or including *Pten*	Small Interstitial Deletion	2	1
19	Including Pten	Large Interstitial Deletion	3	0
19		Large Interstitial Deletion	1	0
X		Telomeric Amplification	1	0
X		Monosomy	1	1
X		Large Interstitial Deletion	1	0

Copy number variations associated with recombination at the TCRβ, TCRγ, TCRα/δ, Skint, IgH, IgLκ and IgLλ loci are omitted for clarity. Where a locus is listed, the feature was observed within this gene, or the feature included this gene, as noted. Where the feature covered multiple genes and was not clearly associated with any one locus, no locus is listed. Although multiple tumours may be listed for any one feature, the precise nature of each copy number change varied between tumours. Where there is more than one of the same type of feature listed for the same chromosome, each feature represents a distinct chromosomal location.

**Table 2:** Comparative Incidence for Genetic Features found in G-TL and C-TL.

	Percent Incidence (Frequency)		
Feature	G-TL	C-TL	*P*-value
Trisomy 14	25% (3/12)	40% (8/20)	0.46
Trisomy 15	67% (8/12)	65% (13/20)	1.00
*Notch1* 5ʹ Deletion	31% (5/16)	49% (49/101)	0.28
*Notch1* PEST Mutation	69% (11/16)	70% (71/101)	1.00
*Notch1* HD Mutation	31% (5/16)	15% (15/101)	0.15
*Ikzf1* LOH	19% (3/16)	27% (27/101)	0.76
*Ikzf1* Mutation	56% (9/16)	42% (42/101)	0.29
*Ikzf1* Mutation/Aberrant Expression	56% (9/16)	56% (57/101)	1.00
*Trp53* Mutation	0% (0/16)	15% (15/101)	0.22
*Trp53* Mutation/Null Expression	0% (0/16)	18% (18/101)	0.13
*Bcl11b* LOH	69% (11/16)	100% (101/101)	0.0001 *
*Pten* Mutation/Null Expression	80% (12/15)	38% (38/101)	0.004 *

*P* values shown are from Fisher’s Exact Test, with differences significant at the 0.05 level marked (*)

**Fig 2:**
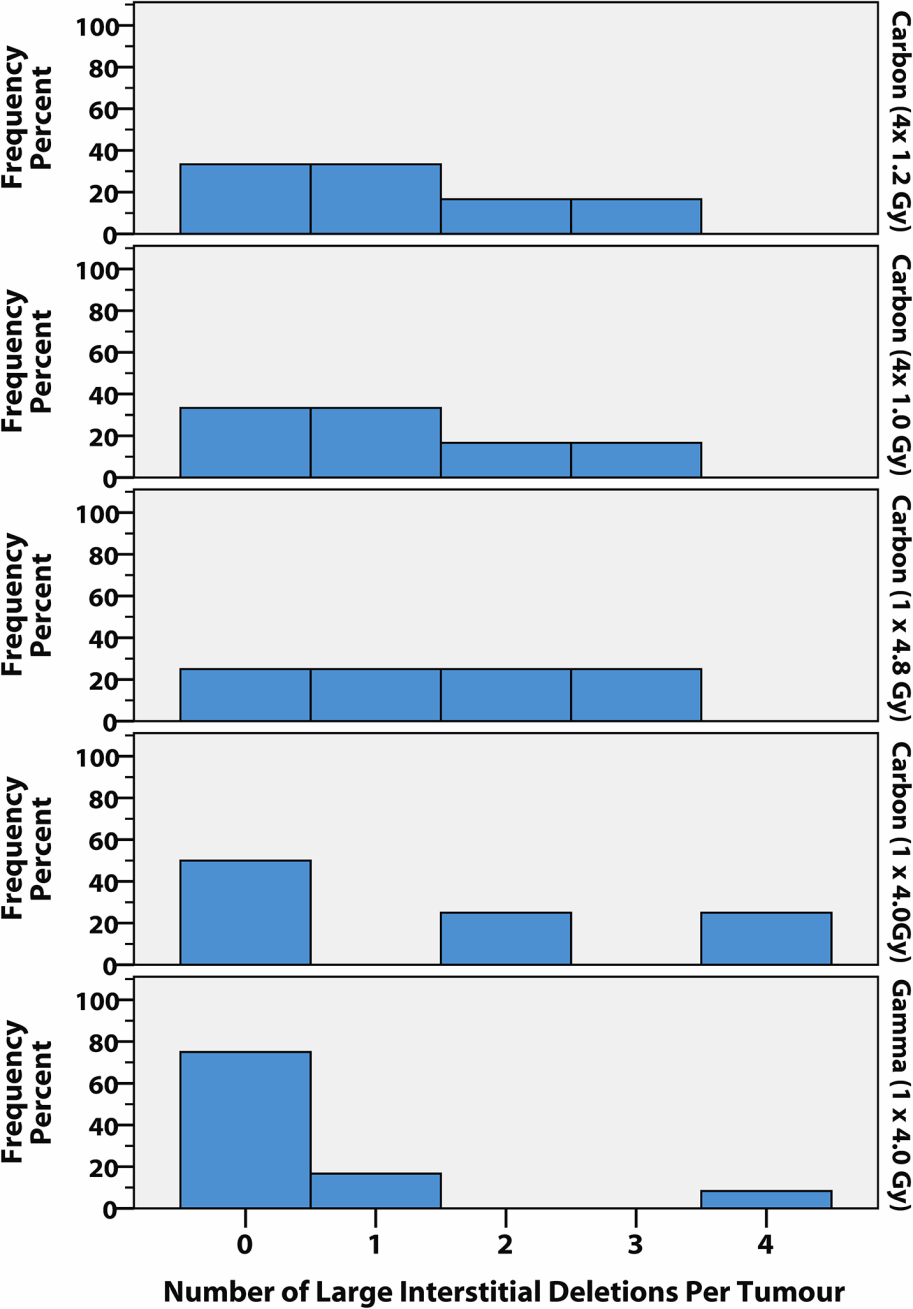
Distribution of large interstitial deletion frequency by irradiation group. The number of large interstitial deletions (>250 kb) per tumour is shown for each treatment group as a percentage of the number of tumours examined by array CGH (*n* = 4–6 per carbon group, *n* = 12 gamma group). The number of tumours analysed per carbon dose group did not permit pairwise comparisons of rank, but the difference between the pooled carbon group (*n* = 20) and the gamma group approaches significance (*P* = 0.053, Mann-Whitney U-Test). Across the four carbon groups the number of TL with at least one large interstitial deletion ranged from 50–75%, while only 25% of G-TL harboured one or more large interstitial deletions (mean of 1.4 large interstitial deletions per C-TL, and 0.5 per G-TL). Interestingly, the single G-TL with 4 large interstitial deletions contained two independent large deletions overlapping over the B cell differentiation gene *Pax5* (the only TL to have *Pax5* involvement) and was the only TL to have a rearranged immunoglobulin light chain gene, which together may point towards a distinct tumour phenotype.

Copy number variations (CNV) in *Notch1* were predominantly observed as a recurrent hemizygous deletion over the 5′ end of the gene (S2 Fig). CNV in the *Cdkn2a*/*Cdkn2b* tandem gene locus were small (maximum size range of 4–210 kb, S3 Fig) except for one tumour with an overlapping compound deletion, and one with a large amplification on chromosome 4 with the breakpoint within *Cdkn2a*. Deletions within the *Ikzf1* and *Pten* loci were also small, ranging from less than 1 kb up to more than 200 kb, and included loss of proximal, terminal or internal regions of the genes, often with common breakpoint regions across multiple TL. In addition, the *Ikzf1*, *Bcl11b* and *Pten* TSG were also included in large interstitial deletion regions (>250 kb) and except in the case of *Pten*, also in deletions extending to the nearest end of the chromosome. The threshold of 250 kb represents the nominal distance covering three sequential probes in the low density genomic tiling of the CGH array, with smaller deletions only detectable in the candidate genes with medium to high density coverage. While there were no significant differences in the frequency of specific recurrent CNVs, comparison of the broader classes of CNV across the genome suggested that large interstitial deletions (>250 kb) were more frequent in C-TL than in G-TL (Fig 2).

### Detailed Analysis of Loci Exhibiting Copy Number Variation

#### *Notch1*

PCR with primers flanking the common deleted region [28] amplified fragments corresponding to the recombination-mediated ‘type 1 deletion’ observed commonly in mouse T cell lymphomas [31, 32]. The deleted alleles are known to produce a transcript from a cryptic 3′ transcription start site producing a constitutively active intracellular Notch. Extension of the Notch type 1 deletion PCR to the full cohort showed that at least 31% of G-TL (5/16) and 49% (49/101) of C-TL (*P* = 0.28, Fisher’s Exact Test) harboured at least one copy of a type 1 deleted allele (not all possible 5′ deletions can be amplified with a single primer set). The *Notch1* PEST domain showed frequent heterozygous insertion/deletion frameshift mutations or nonsense mutations (S5 Table), all predicted to produce truncated NOTCH1 proteins lacking the C-terminal negative regulatory domain, while the HD domain showed a lower frequency of point-mutations or in-frame insertion/deletions (S6 Table), all missense mutations known or predicted to interfere with γ-secretase cleavage [33], with no differences in PEST or HD mutation frequency between C-TL and G-TL (*P* > 0.15, Fisher’s Exact). Mutations in both domains were not mutually exclusive (*P* = 0.59, Fisher’s Exact), but mutations in the PEST domain of *Notch1* were highly associated with tumours positive for the type 1 deletion (*P* < 0.0001, Fisher’s Exact); while conversely, mutations in the HD domain were highly associated with type 1 deletion-negative tumours (*P* < 0.003, Fisher’s Exact). These data support the model that *Notch1* 5′ deletions provide a selective advantage only when paired with a PEST domain mutation, and make HD mutations obsolete (since the domain is not transcribed); and, that HD mutations alone provide a robust increasing in Notch signalling, but confer a hyperactive Notch signal when paired with PEST mutations *in cis* [33, 34]. *Notch1* 5ʹ deletions detected by CGH (S1 Fig) were more frequently associated with tumours that did not harbour trisomy 15 (7/10, 70%) over those with trisomy 15 (5/21, 24%, *P* = 0.021, Fisher’s Exact) suggesting a redundancy between the *Notch1* and *Myc* pathways.

#### *Ikzf1*

**Fig 3:**
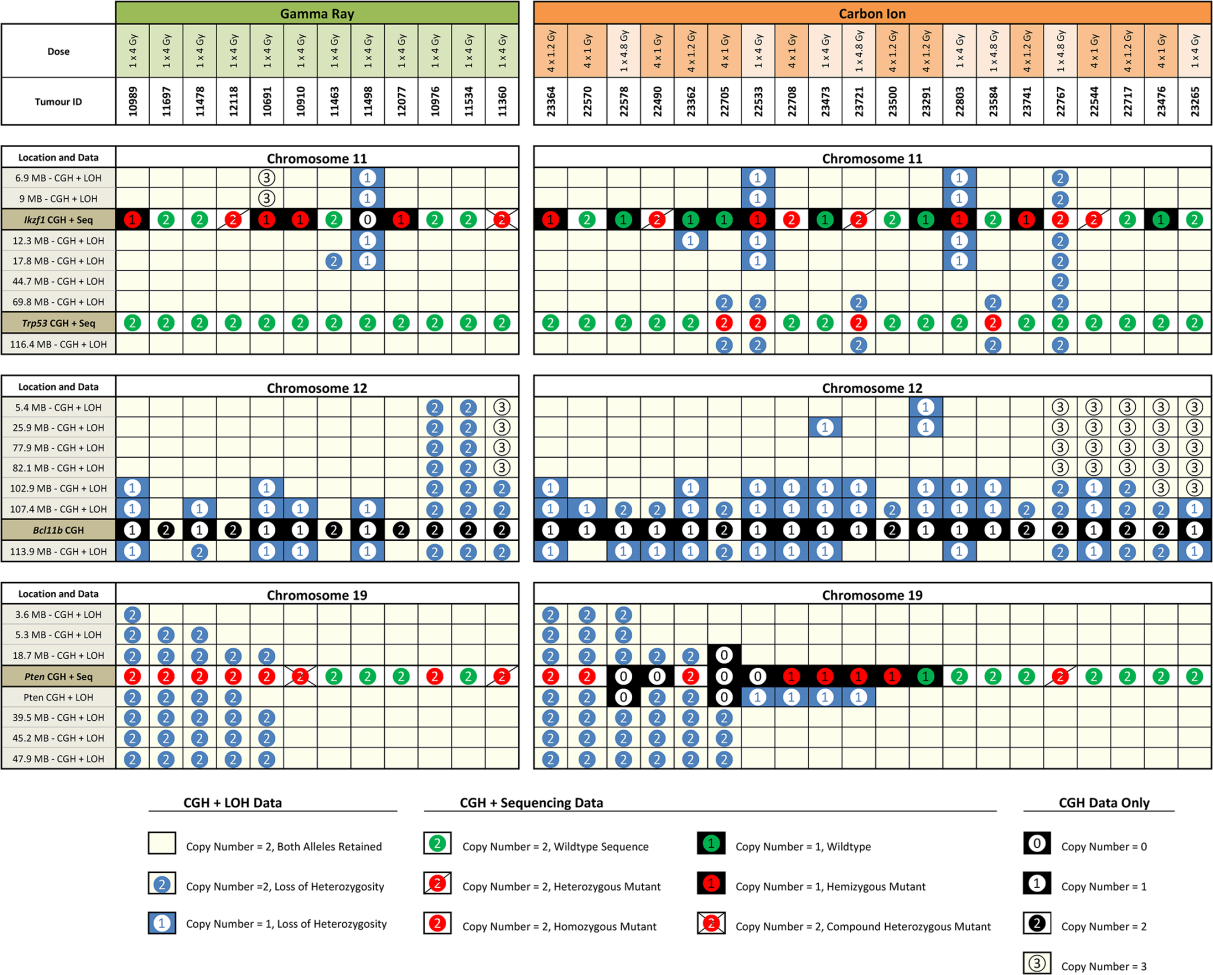
Combined DNA copy number information, loss or retention of heterozygosity and mutation screening. Data for chromosomes 11, 12 and 19, from twelve G-TL and twenty C-TL are shown. Each column represents a single tumour down through all three panels. Columns are ordered manually based on observed groupings. Each row represents either LOH and DNA copy number (CGH) data, DNA copy number data only (*Bcl11b* locus) or DNA copy number and mutation data (*Ikzf1*, *Trp53* and *Pten* loci). Locations noted in each row are from GRCm38 positioning.

Combining the copy number information for the array CGH sub-cohort samples with the results of LOH analysis along chromosome 11 and sequencing of *Ikzf1* transcripts (S7 Table) revealed multiple modes of *Ikzf1* inactivation including: interstitial or centromeric deletion, with or without mutation of the remaining allele; mutation of one allele without change in copy number; or, mutation of one allele which was then duplicated by mitotic recombination along the length of the chromosome (Fig 3). When LOH analysis at two sites flanking the *Ikzf1* locus was performed across the whole cohort, there was no significant difference in LOH frequency between C-TL and G-TL (*P* = 0.76, Fisher’s Exact), nor were there differences in the frequency of aberrant or mutant *Ikzf1* transcript expression. Mutations in both irradiation groups were dominated by non-silent point-mutations in the codons of the zinc finger motifs in the DNA binding or dimerization domains, truncating mutations removing one or both zinc finger regions, or mutations resulting in aberrant isoforms lacking one or more internal exons (S7 Table). Normal *Ikzf1* expression was associated with increased tumour latency compared to TL with *Ikzf1* inactivation (S7 Fig).

#### *Trp53*

Although no copy number variation was observed at the *Trp53* locus in the sub-cohort samples, the LOH analysis extending the length of chromosome 11 revealed a number of cases of recombination-mediated telomeric LOH (without copy number loss) in C-TL (Fig 3), which were found to be associated with homozygous mutations in *Trp53*. Mutations in *Trp53* (S8 Table) were observed in 15% of the whole C-TL cohort but in none of the sixteen G-TL (*P* = 0.22, Fisher’s Exact), and an additional 3% of C-TL lacked *Trp53* expression. As might be expected being co-located on chromosome 11, *Trp53* mutations and LOH at the *Ikzf1* locus showed a significant association (*P* = 0.022, Fisher’s Exact). Analysis of TL latency showed divergent associations with *Trp53* status, with mutant *Trp53* associated with increased latency, while lack of *Trp53* expression decreased tumour latency (S6 Fig) compared to *Trp53* wildtype TL.

#### *Bcl11b*

**Fig 4:**
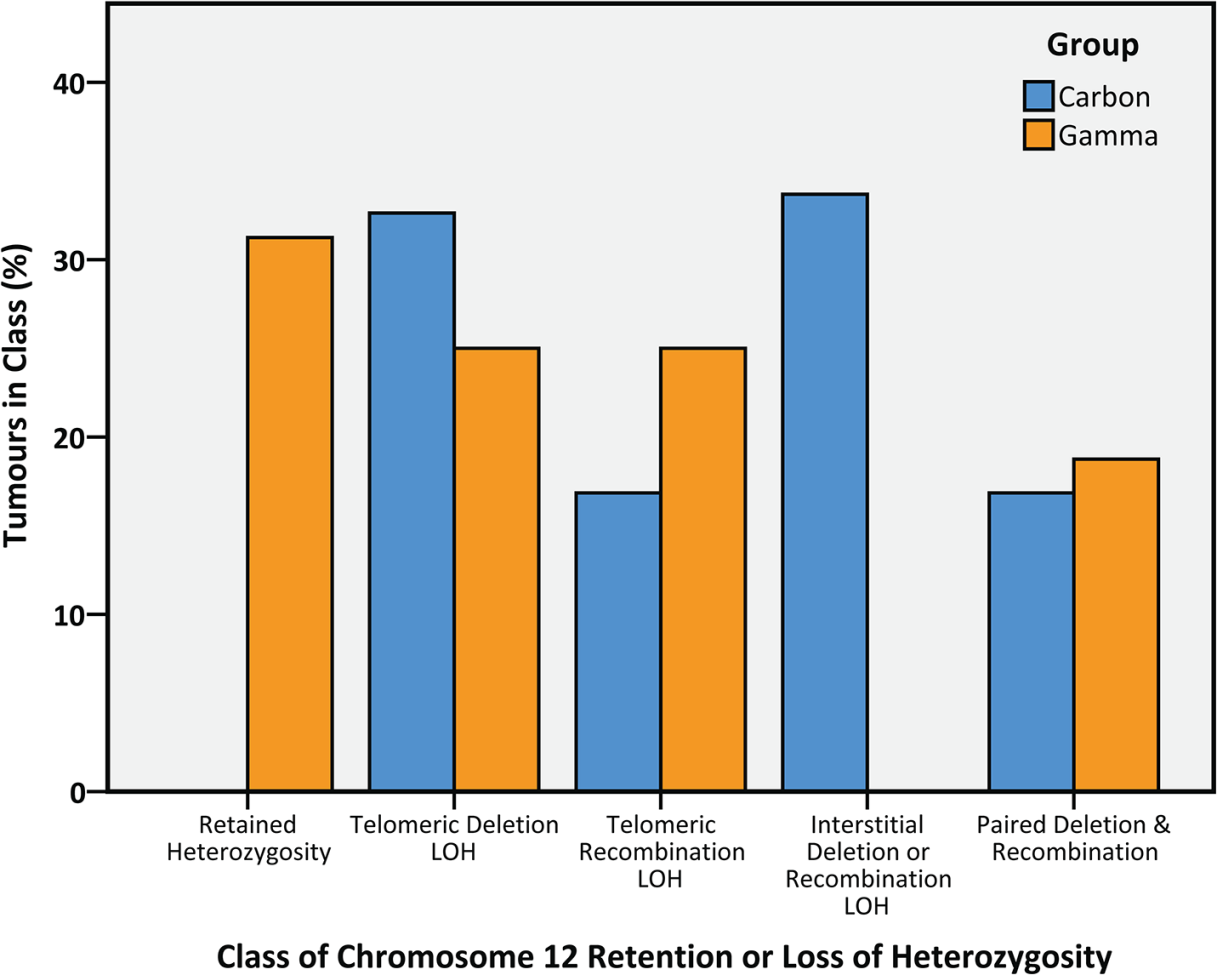
Classification of tumours by mechanism of retention/loss of heterozygosity at markers proximal and distal to *Bcl11b* on chromosome 12. C-TL (*n* = 93) and G-TL (*n* = 16) were classified according to the LOH results at D12Mit132 (Proximal to *Bcl11b*) and D12nds2 (Distal to *Bcl11b*) on chromosome 12 combined with DNA copy number results from array CGH and/or DNA copy number RQ-PCRs at D12Mit132, D12nds2 and within *Bcl11b* (the remaining 8 C-TL samples had insufficient DNA for copy number screening). The frequencies of Telomeric Deletion LOH (deletion at both sites), Telomeric Recombination LOH (recombination at both sites) and Paired Deletion & Recombination (both mechanisms in either order) were similar for both carbon and gamma groups, yet the overall distribution of the classes was significantly different between carbon and gamma groups (P<0.0001, Pearson’s Chi-Squared) since although 5/16 gamma tumours (31%) retained both alleles either side of *Bcl11b* (Retained Heterozygosity), 0/93 carbons tumours retained both alleles (*P* = 0.0001, Fisher’s Exact). In contrast, 32/93 carbon tumours (34%) lost one allele at D12Mit132 (upstream of *Bcl11b*) and yet retained both alleles towards the telomere at D12nds2 (Interstitial Deletion or Recombination LOH) while this was not observed (0/16) in gamma tumours (*P* = 0.003, Fisher’s Exact).

The combination of DNA copy number information and LOH analysis extending along the length of chromosome 12 (Fig 3) revealed that *Bcl11b* –located towards the telomere of chromosome 12 –was almost universally vulnerable to loss by deletion of the distal segment of the chromosome, LOH by mitotic recombination, as well as by large interstitial deletions including the *Bcl11b* locus. Previous investigations have demonstrated that *Bcl11b* is haploinsufficient for its tumour suppression effects [35], however in this study, mitotic recombination and non-disjunction events were also apparent at high frequency. Since *Bcl11b* mutations were not investigated here, it is unclear whether duplication of putative mutant alleles or removing wild-type alleles at the *Bcl11b* locus is the driver of such events or whether mutations in other loci (such as the downstream *Akt1*, MGI: 87986), or even amplification of centromeric loci (as observed in both C-TL and G-TL) are responsible for the selective pressure. Extending the LOH analysis at two sites flanking the *Bcl11b* locus to the whole cohort revealed LOH on one or both sides in 100% of C-TL but only 69% of G-TL (*P*<0.001, Fisher’s Exact). Using a real-time quantitative PCR assay to measure DNA copy number near the two LOH flanking sites and within *Bcl11b* itself across the whole cohort (Fig 4) gave further evidence to support the hypothesis that the frequency of interstitial deletion (as well as interstitial recombination) events was more frequent in C-TL, and that this additional mechanism may explain the increased penetrance of *Bcl11b* involvement. In fact, LOH flanking *Bcl11b* was a strong predictor of TL latency within the G-TL cohort (S4 Fig) and could account for much of the difference in latency between the 1 × 4 Gy carbon and gamma exposures (S5 Fig).

#### *Pten*

DNA copy number information was combined with LOH analysis extending along the length of chromosome 19 and sequencing of *Pten* transcripts (Fig 3). Unlike the *Ikzf1* and *Bcl11b* loci, the *Pten* locus was insulated from loss of chromosome ends but was still susceptible to intragenic deletions within the *Pten* gene, and (in the C-TL) large interstitial deletions, with the remaining *Pten* allele frequently mutated. The LOH data also revealed frequent duplication of mutated *Pten* alleles by mitotic recombination involving most or all of chromosome 19. Analysis of an LOH marker inside the *Pten* gene across the whole cohort showed the same LOH frequency in C-TL and G-TL (31%). Yet, the frequency of *Pten* abnormalities was significantly higher in G-TL (80%) than in C-TL (38%, *P* = 0.004, Fisher’s Exact) due to an increased frequency of *Pten* mutations and lack of *Pten* expression. Mutations were usually non-silent point-mutations, aberrant transcripts with missing exons or insertion/deletions introducing premature stop codons (S9 Table).

### Comprehensive Analysis

**Fig 5:**
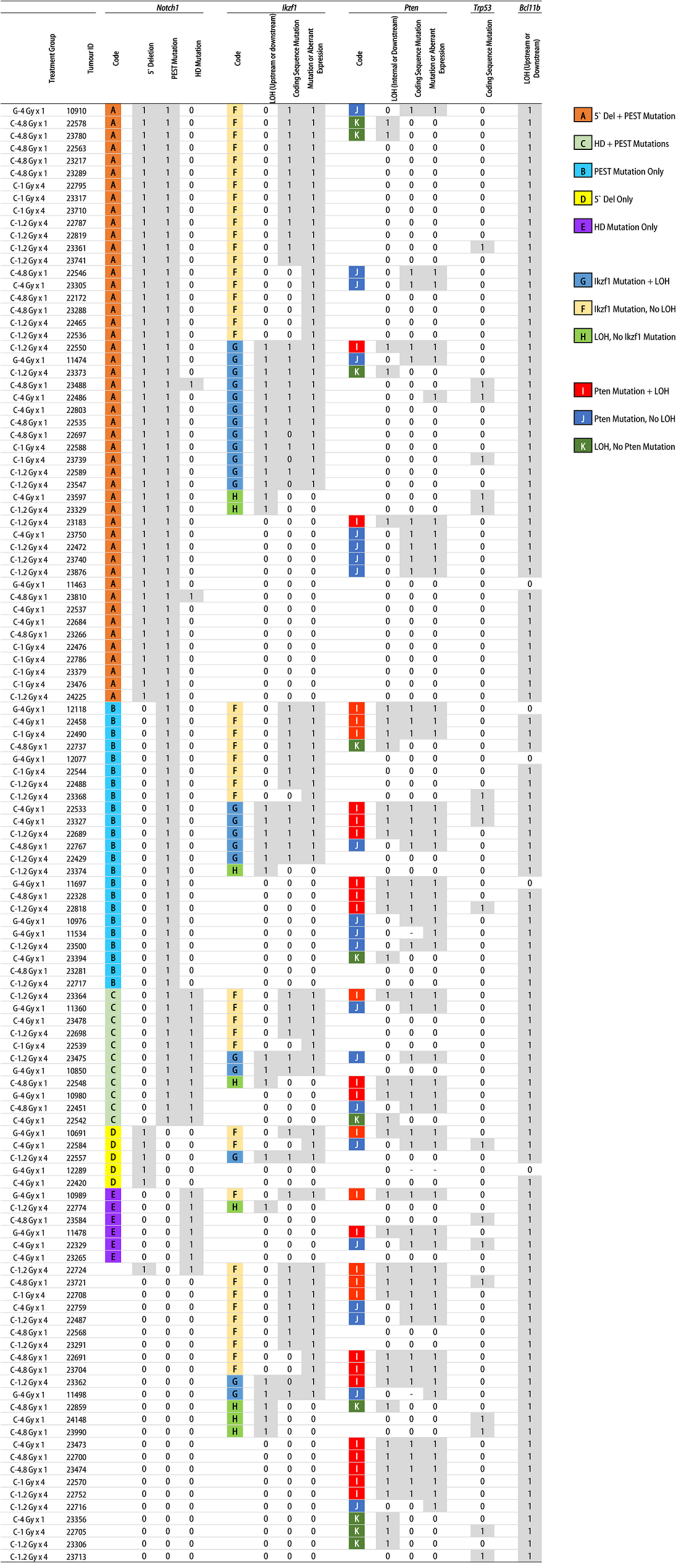
Patterns of Oncogene Activation and TSG Inactivation. All 117 tumours are sorted by the presence (1) or absence (0) of specific aberrations in *Notch1*, *Ikzf1*, *Pten*, *Trp53* and *Bcl11b*, with (-) denoting an unclear result. Patterns of activation/inactivation are highlighted by letter codes as shown in the key, for *Notch1* (A-E), *Ikzf1* (F-H) and *Pten* (I-K). Almost complete LOH at the *Bcl11b* locus can be observed, along with high frequency of *Notch1* aberrations. *Pten* aberrations, and particularly homozygous *Pten* mutations, are more frequently associated with tumours not carrying the combined 5′ Deletion and PEST mutation in *Notch1*.

Comparison of all the variables confirms that the previously established hallmarks of radiation-induced TL are frequent in both G-TL and C-TL (Table 2), with the exception of *Bcl11b* LOH and *Pten* mutation/null expression which were more frequent in C-TL and G-TL, respectively, and *Trp53* mutations/null expression which were infrequent and only found in C-TL (which had a larger sample size). There were no significant differences in the incidence of any of these tested features between the four carbon ion treatment groups (Pearson’s Chi-squared test), by total carbon dose (Fisher’s Exact test, pooling single exposure and fractionated groups) or between single and fractionated carbon (Fisher’s Exact test, pooling 4.0 and 4.8 Gy groups). Relating the data on LOH, coding sequence mutations and/or aberrant expression of *Notch1*, *Ikzf1*, *Pten*, *Trp53* and *Bcl11b* for each tumour allows a comprehensive analysis of the mechanisms of oncogene activation and TSG inactivation (Fig 5). The ubiquity of *Bcl11b* LOH can be clearly seen, as can the dominant role of activated Notch signalling. The loss of PTEN function, particularly the inactivation of both alleles, is observed primarily in tumours without the hyper-activated combination of *Notch1* 5′ deletion plus PEST domain mutation. Additional loss of *Ikzf1* in addition to Notch hyper-activation seems to negate the need for PTEN disruption at the genetic level.

## Discussion

Very little is known about the relative carcinogenicity of carbon ion radiation. Here, TL was more frequent and manifested earlier after exposure to a single dose of 4 Gy carbon ions compared to a single 4 Gy dose of gamma rays. There was evidence of reduced TL risk and increased TL-free lifespan with carbon ion fractionation at 4 Gy total dose, but not at 4.8 Gy total dose. It is likely that above a certain total dose threshold, the effect of fractionation diminishes with different kinetics for leukaemias, lymphomas and solid tumours resulting in a complex balance between the competing risks.

### Carbon ion- and gamma ray-induced TL share the same set of activated/inactivated genes

The genomic loci most frequently associated with DNA copy number variation in the radiation-induced tumours tested here were the same as those previously identified in photon radiation-induced tumours [17–20, 36]. These reflect the principal causal genes previously established as underlying the development of both human T-ALL and mouse TL, and are part of a complex network orchestrating normal T lymphocyte development. Loss of *Bcl11b* alleles by deletion or recombination was nearly complete in both C-TL and G-TL, testament to its key role in enabling TL expansion. Clonal expansion of cells with LOH at the *Bcl11b* locus has been reported as being one of the earliest detectable changes (along with *Myc* amplification) in the thymus post-irradiation but prior to the onset of lymphoma [37]. The presence of *Notch1* Type 1 deletion-bearing cells in the normal mouse thymus [38] suggests that activating *Notch1* deletions in the TL could result from selective expansion of cells with pre-existing spontaneous deletions, yet other evidence indicates that the recombination-mediated deletions are a later spontaneous event in the carcinogenic sequence [37, 39], with no increase in deletion frequency in TL from irradiated compared to unirradiated mice [31]. Since studies of large panels of T-ALL have failed to find equivalent recombination-mediated deletions in the human *NOTCH1* gene [32], this represents a mouse-specific tumour susceptibility that may contribute to their increased vulnerability to radiation-induced T cell tumours; though, the finding of a NOTCH1 Type 2 activating deletion (not recombination mediated) in a T-ALL patient [40] demonstrates that the mechanism has *prima facie* relevance to human cancers. Alternate pathways of activating Notch signalling, such as *FBXW7* mutations (Gene ID: 55294) are seen in paediatric T-ALL [41], and tumours arising in *Fbxw7* ^+/-^ mice do not show *Notch1* mutations or type 1 deletions [28] confirming the existence of redundant pathways for achieving tumour-permissive Notch signalling in both mice and humans.

Loss of PTEN has been shown to interfere with normal *Ikzf1* splicing [42], and since IKZF1 and NOTCH1 compete for repression and activation of an overlapping set of genes [43, 44], even transient changes in expression of the various functional and dominant-negative IKZF1 isoforms during development (even without mutation) could permit a self-reinforcing Notch signalling state to gain a foothold [45]. Furthermore, since IKZF1 can bind both the canonical and cryptic internal *Notch1* promoter [46], the important role of *Ikzf1* loss/mutation in mouse TL as opposed to human T-ALL (where the importance of its role is contested) [47] may again be linked to species-specific mechanisms for establishing Notch signalling [44, 48]. Notch signalling may remove the selective pressure for *Trp53* mutations, by directly blocking TRP53-dependent apoptosis [45, 49]; and although increased Notch signalling (in *Fbxw7* ^+/-^ mice) has been shown to be synergistic with *Pten* heterozygosity for radiation-induction of TL [50], as observed in this study, strong Notch signalling from a combined 5′ deletion and PEST mutation may negate the need for *Pten* inactivation [37]. Although the high frequency of *PTEN* mutations in human cancers is often cited, there has been some debate over the extent of its involvement in T-ALL due to *PTEN* mutations being observed in a minority of human T cell cancers. Yet more comprehensive analyses of the whole PTEN pathway in paediatric T-ALL have revealed disruption of the PTEN-PI3K-AKT pathway in almost half of the cases examined [51].

### The effects of carbon ions at low LET are more similar to photon irradiation than to carbon ions at high LET

A variety of *in vitro* studies provide data about the relative effects of carbon ion irradiation on cell killing, mutation frequency and DNA repair kinetics, although it is important to distinguish which effects are restricted to high LET exposures. For radiation-induced chromosome breaks and cell survival, carbon ions at low LET (13 keV.μm^-1^) were more similar to the X-ray exposure than the increased damage observed with carbon ions at higher LET (75 keV.μm^-1^) [52]; however, even carbon ions at higher LET (70 keV.μm^-1^) have been shown experimentally to be more similar to the reference X-ray exposures in terms of initial DNA breaks than the heavier ion species (and higher LETs) tested [53]. The localised energy deposition from heavy ions is thought to increase the probability of isochromatid-type DNA breaks in G2 cells, consistent with the greater number of track intersections required to produce such events with low LET radiations [54]. Yet similar levels of potentially-lethal damage repair after 13 keV.μm^-1^carbon ions and 200 kVp X-rays have been observed, in contrast to significantly reduced repair observed after 75 keV.μm^-1^carbon ions [55]. This is consistent with findings that radiation-induced free-radical damage is still significant for carbon at low LET but not at higher LET [56]. Modelling suggests that DNA DSB induced by carbon ion traversal at low LET in the range used in this experiment are almost completely due to single-event DSB rather than randomly coincident single-strand breaks (SSB), just as for photons in the therapeutic dose range [57]; while enhancement of DNA DSB incidence due to inter-track co-localised SSB is predicted for carbon ions at LET above 100 keV.μm^-1^. Furthermore, a local effect model which considers clustered damage not in terms of ‘locally multiply damaged sites’ [58] but on the larger scale of ‘regionally multiply damaged sites’ whereby two or more DNA DSB within a single DNA loop domain can result in the loss of large stretches of DNA predicts the increasing incidence of such regional clustered damage from single carbon ion tracks of increasing LET [59]. If the unique lesions of interest for carbon ion-induced carcinogenesis are such large interstitial deletions occurring in surviving cells, it is possible that the RBE for cancer induction for carbon ions at lower LET are larger than what might be predicted from RBE models based on relative cell killing.

The enhanced cell killing effect of carbon ions compared to gamma rays is dependent on increasing LET [60], with indications that NHEJ-mediated repair of low LET–type DNA damage increases the survival probability of irradiated cells. Cells have been shown to be most sensitive to cell killing from carbon ion irradiation during the G2/M phase at both low and high LET [61]. Although the carbon ion exposures at intermediate (50.8 keV.μm^-1^) and high LET (76.5 keV.μm^-1^) also caused maximal increases in mutation frequency during G2/M phase, surprisingly, the low LET carbon exposure (13 keV.μm^-1^) showed no such hypersensitivity to mutation during G2/M phase. It was postulated that while homologous recombination is able to complete error-free repair of low LET-type damage induced during G2/M phase, the complex high LET-type damage is more likely to be misrepaired by homologous recombination [61].

### The phenotype of radiation-induced TL is largely determined by selection for canonical mutations rather than by hallmarks of radiation-induced damage

In both the C-TL and G-TL, loss of TSG and activation of oncogenes occurred via a variety of mechanisms, which were dependent on both the chromosomal location of the locus, and gene-specific factors such as haploinsufficiency and the selection of dominant-negative phenotypes. The small number of TSG disruptions associated with TL [62] provides immense selective pressure to inactivate most of these particular genes in all TL analysed, resulting in a natural homogeneity which is in contrast to the complexity and heterogeneity which has recently been observed in human T-ALL [63]. Most clonal lesions present in tumours from irradiated animals are not caused directly by radiation even if the tumours themselves are ‘radiation-induced’, with many if not most lesions arising independently of radiation later during tumour progression [64]. Given the observed prevalence here of simple DNA mutations including point-mutations and insertion/deletion mutations at homopolymer runs within the gene coding sequences of TSGs, a scenario can also be envisaged whereby radiation results in loss of a wildtype allele by deletion or mitotic recombination, revealing a pre-existing spontaneous mutation which might have conferred little selective advantage when in a heterozygous state. There is also evidence that existing TSG mutations which may not confer a strong selective advantage within a normal tissue environment, can become highly advantageous with reduced competition in the tissue environment following irradiation [65]. Here, differences in the pattern of TSG inactivation between C-TL and G-TL are likely due to a cascading effect from the differential probability of perhaps one keystone event (e.g. increased physical chance of *Bcl11b* loss by interstitial deletion/recombination) which due to the co-operative/redundant nature of the pathways means that each successive mutation/lesion defines the selective pressure for the next event(s) driving tumours towards phenotypes reflecting one or more oncogenic networks.

Against this background, it is reasonable to expect that an additional carbon radiation-induced deletion/recombination mechanism would not result in a novel kind of TL with a highly divergent genetic aetiology, nor in a subset of induced TL showing ‘carbon-type’ mutations at all disrupted loci. Rather, an increase in the probability of producing a TL-initiating lesion would be expected (as seen in the increased incidence of C-TL) in addition to a modest shift in the distribution of lesion types (as also observed here for large interstitial deletions by array CGH and for interstitial deletion/recombination at the *Bcl11b* locus). The consideration of interstitial deletion/recombination events in two (or more) size classes is supported by other studies showing that micro-deletions are a general feature of human T-ALL [66], and that recurrent focal deletions (such as observed here in *Notch1*, *Cdkn2a*, *Ikzf1* and *Pten*) are likely the result of illegitimate recombination events [67] rather than the repair of radiation-induced DNA damage.

If much of the tumour-inducing action of radiation is physiological (ablation of the thymus, followed by rapid, irregular re-population from a limited pool of potentially-damaged stem/progenitor cells) rather than directly genetic/mutational, it might be expected that some carbon radiation-specific genetic lesions could be found at non-causal genomic locations, i.e. as passenger rather than driver mutations. It is interesting that many of the large interstitial deletions observed in C-TL were unique and affected only genes not implicated previously in lymphomagenesis and/or genes not generally considered as initiating events (even if they may have provided some selective advantage). These factors, particularly a large role for recombination-induced TSG inactivation and the fact that the interstitial deletions observed in C-TL were usually less than 2 Mb in size and almost all <5 Mb (and hence cytogenetically cryptic) give caution to the use of cytogenetic assays to assess the relative carcinogenic risk of heavy ions, since the relevant mechanisms may not be suitably measured. Despite the obvious differences between TL in inbred mice and human T-ALL, the observation of a class of DNA/chromosomal lesion which seems to be more prevalent in carbon ion-induced tumours would appear to be directly relevant to the risks associated with radiotherapy-induced carcinogenesis in humans, for a range of tissues and their obligate TSG. Though further work will be required to establish increased production of interstitial deletions as a *bona fide* mechanism of carbon ion-induced carcinogenesis, it is biologically and physically plausible that even the lower LET radiation in in the normal tissue ahead of the target volume during high LET carbon radiotherapy could pose a greater carcinogenic risk per unit dose than similar non-target radiation deposited during photon radiotherapy.

## Supporting Information

S1 ARRIVE Checklist The ARRIVE Guidelines Checklist. (PDF)

S1 Dataset Full Tabulation of Tumour Data for LOH, Gene Expression and Mutation Sequencing. (XLSX)

S1 Fig Occurrence of Rearrangements, Specific Deletions or Specific Chromosomal Aberrations as Detected by Array CGH. For each TL, (1) and (0) represent the presence or absence, respectively, of the features shown in the column headings. Other values are as defined here: (2) Centromeric deletion with breakpoint at *TCRa/d* locus; (3) Amplification of region immediately upstream of *IgH* locus; (4) interstitial deletion including *IgLκ* locus; (5) Internal deletion within *Notch1* (not recurrent 5′ deletion); (6) *Ikzf1* internal amplification.
(TIF)

S2 Fig Frequent Deletions in 5′ region of *Notch1* Detected by Array CGH. Deletions over the 5′ end of the *Notch1* gene (bottom of gene in the image) were frequent in TL from gamma- and carbon-irradiated mice, with most corresponding to a specific 11.8 kb deletion as well as one TL with a large internal deletion and one with a smaller 3′ deletion (top of the gene in the image). Lines show the moving average of log_2_ tumour-to-reference DNA copy number ratio (deletions to the left, amplifications to the right), with TL from gamma-irradiated mice in red, and those from carbon-irradiated mice in blue. Red and blue solid bars denote automatic aberration detection by the analysis software.
(TIF)

S3 Fig Frequent Deletions of *Cdkn2a/Cdkn2b* Detected by Array CGH. Deletions over the *Cdkn2a*/*Cdkn2b* locus were small, focal and affecting only the tandem gene locus. Lines show the moving average of log_2_ tumour-to-reference DNA copy number ratio (deletions to the left, amplifications to the right), with TL from gamma-irradiated mice in red, and those from carbon-irradiated mice in blue. Red and blue solid bars denote automatic aberration detection by the analysis software.
(TIF)

S4 Fig Lack of LOH Flanking *Bcl11b* is Associated with Increased TL Latency. Within the G-TL cohort, there was a significant increase in TL latency (*P* = 0.001, Log-Rank) associated with retention of both *Bcl11b* alleles, with an increase in median lifespan of 162 days for mice with TL that did not harbour *Bcl11b* LOH (*n* = 5, median 326 days) compared to those that did (*n* = 11, median = 164 days).
(TIF)

S5 Fig Difference in TL Latency between Gamma and Carbon (at 1 x 4 Gy exposure) is Associated with LOH flanking *Bcl11b*. When the mice without LOH flanking *Bcl11b* are separated (*n* = 5), the remaining TL in mice treated with 1 x 4 Gy show no difference in latency between gamma ray (*n* = 11) or carbon ion irradiation (*n* = 21, *P* = 0.96, Log-Rank).
(TIF)

S6 Fig Difference in TL Latency is Associated with *Trp53* Status. Across the whole TL cohort, TL with mutant *Trp53* expression (*n* = 15) showed an increased tumour latency (*P* = 0.018, Log-Rank), while TL with null *Trp53* expression (*n* = 3) showed decreased tumour latency (*P* = 0.09, Log-Rank) compared to *Trp53* wildtype TL (*n* = 99). This effect is likely a distinction between rare, early *Trp53* loss events which were produced directly by the radiation which lead to rapid tumour progression, the majority of tumours which have no selective pressure to acquire *Trp53* mutations due to alternate events which bypass *Trp53*, and more indolent tumours which acquire *Trp53* mutations late due to sustained selection pressure.
(TIF)

S7 Fig Difference in TL Latency is Associated with *Ikzf1* Status. Across the whole TL cohort, TL with normal *Ikzf1* expression (*n* = 55) had significantly increased tumour latency than TL with mutant sequence (*n* = 66), low levels and/or aberrant splicing (*P* = 0.037, Log-Rank).
(TIF)

S1 Methods Extended Materials and Methods. (PDF)

S1 Table TL Incidence and Characteristics of Tumours Included in the Study. The crude incidence represents the number of mice diagnosed with TL at necropsy out of the total number of mice per group, without correction for competing risks. The body weight at the time of necropsy was significantly higher in mice receiving fractionated carbon irradiation compared to the same doses as a single exposure (*P* < 0.036, Bonferroni-Corrected Independent Samples T Test, two-tailed) an indication of the sparing effect on normal body development.
(PDF)

S2 Table Distribution of Thymus and Spleen Mass for Tumours Included in the Study. The threshold for low/high thymus weight (300 mg) represented the lowest quartile, and the threshold for high/low spleen weight (200 mg) seemed to separate a bimodal distribution likely indicating splenic infiltrating and non-infiltrating tumours. There was no significant association between the treatment group and the thymus/spleen organ weight status at necropsy *(P* = 0.40, Pearson’s Chi-squared Test), but there was a strongly significant negative correlation between thymus and spleen weight across the whole cohort (Pearson Correlation Coefficient = -0.33, *P* = 2.5×10^−4^) perhaps indicative of a tumour-specific preference for expansion in the thymus or the periphery.
(PDF)

S3 Table Distribution of Immunophenotypes between Carbon Ion Treatment Groups. Here, 35 sequential carbon ion-induced TLs were examined at the time of necropsy for cell surface expression of the CD4, CD8, CD90 and CD45R cell surface antigens. All of the tumours were CD90+ and CD45R negative, indicative of T cell origin. The immunophenotypes included not only the standard CD4/CD8 double positive, double negative and single positive subtypes, but in addition, included tumours where one or both antigens were expressed along a continuum from low to high expression level (CD4+/- or CD8+/-). Three tumours also displayed two or more distinct immunophenotypes (as opposed to the aforementioned indistinct phenotypes). There was no significant association between treatment group and immunophenotype (*P* = 0.72, Pearson’s Chi-squared Test); however, the low numbers make it difficult to draw any firm conclusions.
(PDF)

S4 Table Details of Sub-Cohort of TL for Array-based CGH Analysis. (PDF)

S5 Table Summary of *Notch1* PEST Domain Sequence Mutations. All *Notch1* PEST domain mutations are predicted to cause truncation by a direct nonsense mutation or a frameshift mutation with downstream premature stop codon.
(PDF)

S6 Table Summary of *Notch1* HD Domain Sequence Mutations. All *Notch1* HD Domain mutations are predicted to produce in-frame codon deletions, insertions or substitutions.
(PDF)

S7 Table Summary of *Ikzf1* Protein Coding Sequence Mutations. (PDF)

S8 Table Summary of *Trp53* Protein Coding Sequence Mutations. All *Trp53* mutations were found in carbon ion-induced tumours.
(PDF)

S9 Table Summary of *Pten* Protein Coding Sequence Mutations. (PDF)
