# Supplementary figures and images for: Genetic Analysis of T Cell Lymphomas in Carbon Ion-Irradiated Mice Reveals Frequent Interstitial Chromosome Deletions: Implications for Second Cancer Induction in Normal Tissues during Carbon Ion Radiotherapy
Source: PLoS One. 2015 Jun 30;10(6):e0130666. doi: 10.1371/journal.pone.0130666 (PMC4488329; doi:10.1371/journal.pone.0130666)

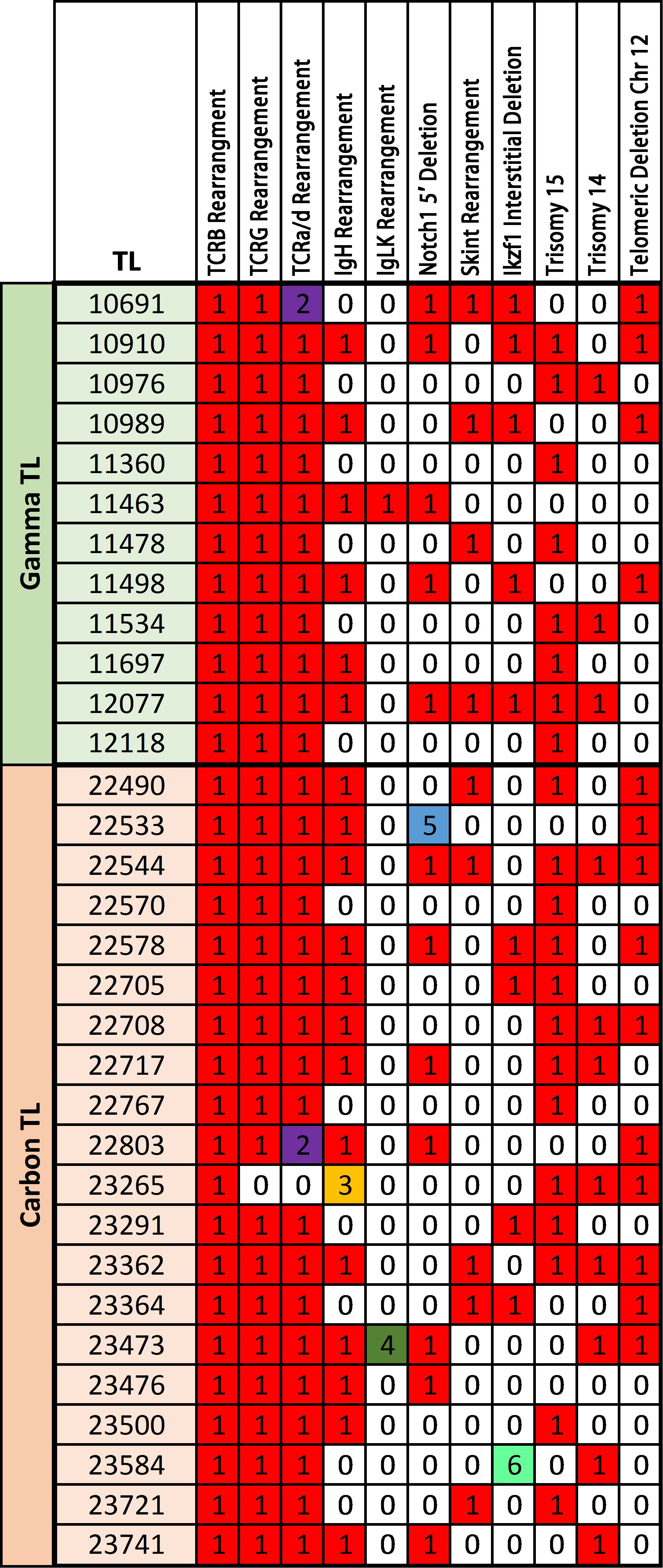

Supplement: S1 Fig — For each TL, (1) and (0) represent the presence or absence, respectively, of the features shown in the column headings. Other values are as defined here: (2) Centromeric deletion with breakpoint at TCRa/d locus; (3) Amplification of region immediately upstream of IgH locus; (4) interstitial deletion including IgLκ locus; (5) Internal deletion within Notch1 (not recurrent 5′ deletion); (6) Ikzf1 internal amplification. (TIF) [file pone.0130666.s003.tif]

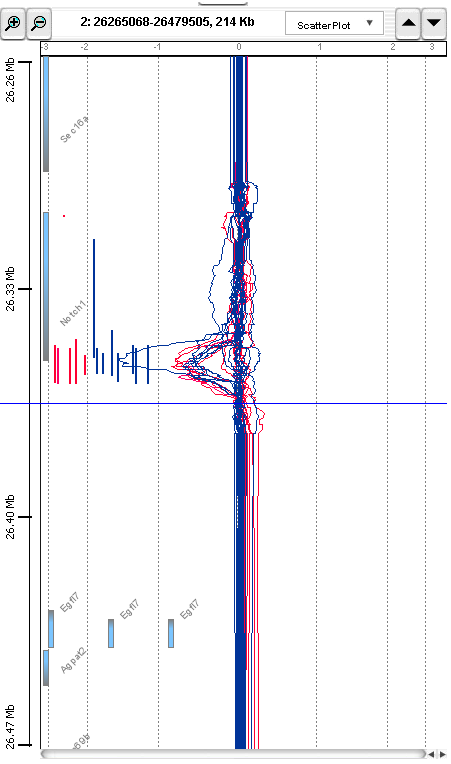

Supplement: S2 Fig — Deletions over the 5′ end of the Notch1 gene (bottom of gene in the image) were frequent in TL from gamma- and carbon-irradiated mice, with most corresponding to a specific 11.8 kb deletion as well as one TL with a large internal deletion and one with a smaller 3′ deletion (top of the gene in the image). Lines show the moving average of log2 tumour-to-reference DNA copy number ratio (deletions to the left, amplifications to the right), with TL from gamma-irradiated mice in red, and those from carbon-irradiated mice in blue. Red and blue solid bars denote automatic aberration detection by the analysis software. (TIF) [file pone.0130666.s004.tif]

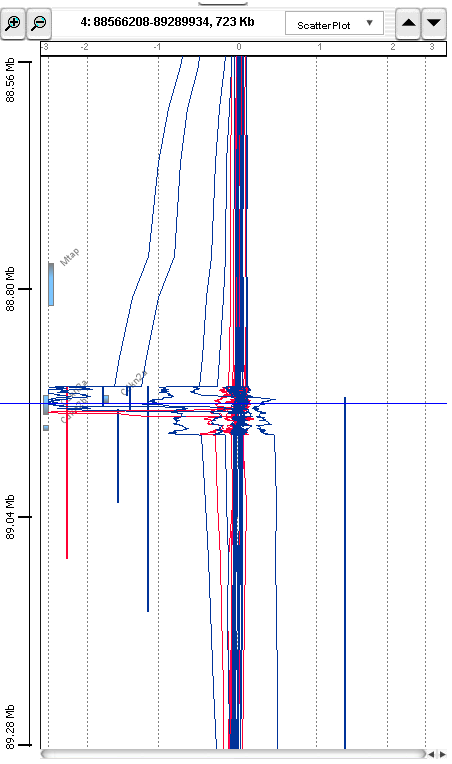

Supplement: S3 Fig — Deletions over the Cdkn2a/Cdkn2b locus were small, focal and affecting only the tandem gene locus. Lines show the moving average of log2 tumour-to-reference DNA copy number ratio (deletions to the left, amplifications to the right), with TL from gamma-irradiated mice in red, and those from carbon-irradiated mice in blue. Red and blue solid bars denote automatic aberration detection by the analysis software. (TIF) [file pone.0130666.s005.tif]

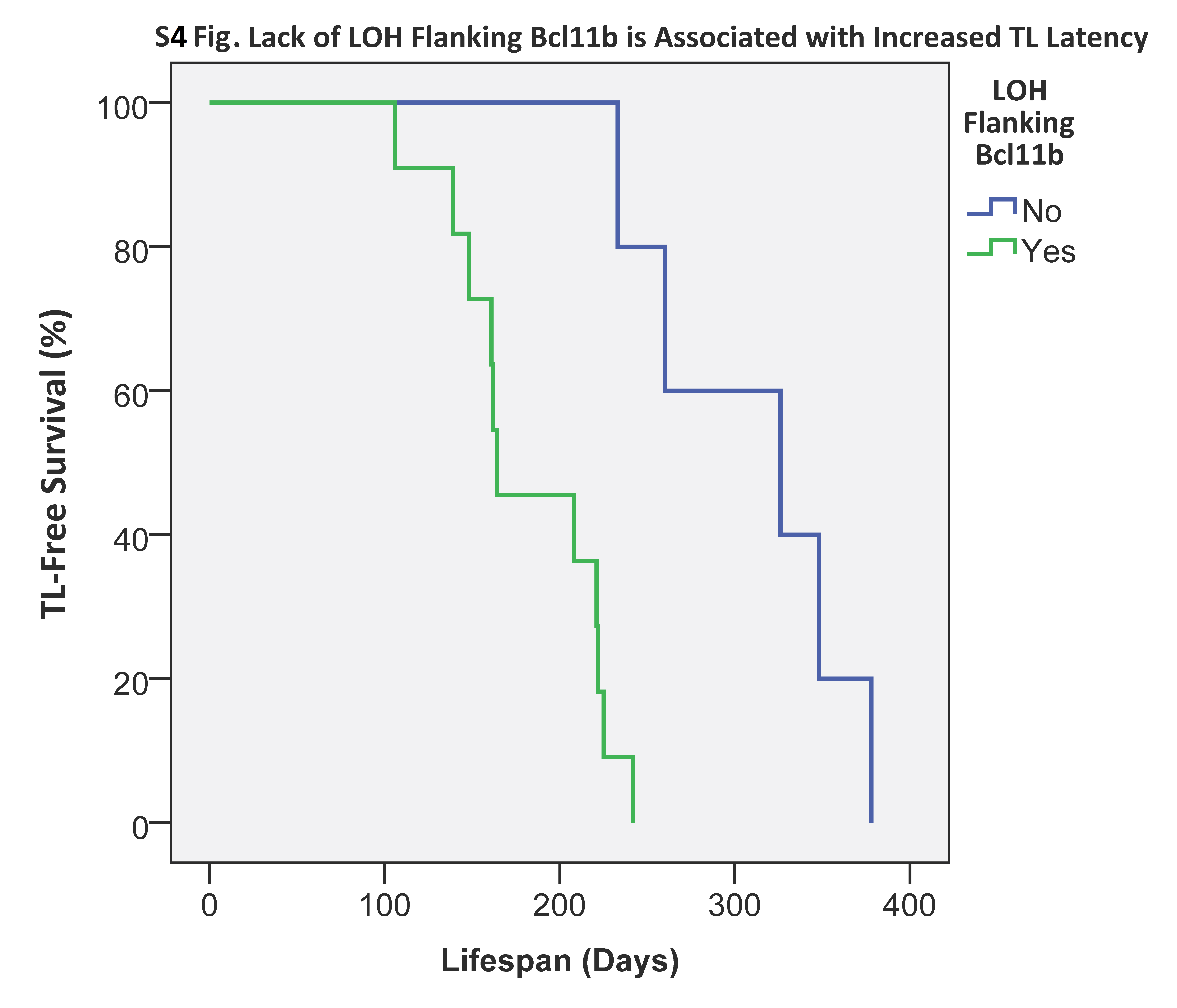

Supplement: S4 Fig — Within the G-TL cohort, there was a significant increase in TL latency (P = 0.001, Log-Rank) associated with retention of both Bcl11b alleles, with an increase in median lifespan of 162 days for mice with TL that did not harbour Bcl11b LOH (n = 5, median 326 days) compared to those that did (n = 11, median = 164 days). (TIF) [file pone.0130666.s006.tif]

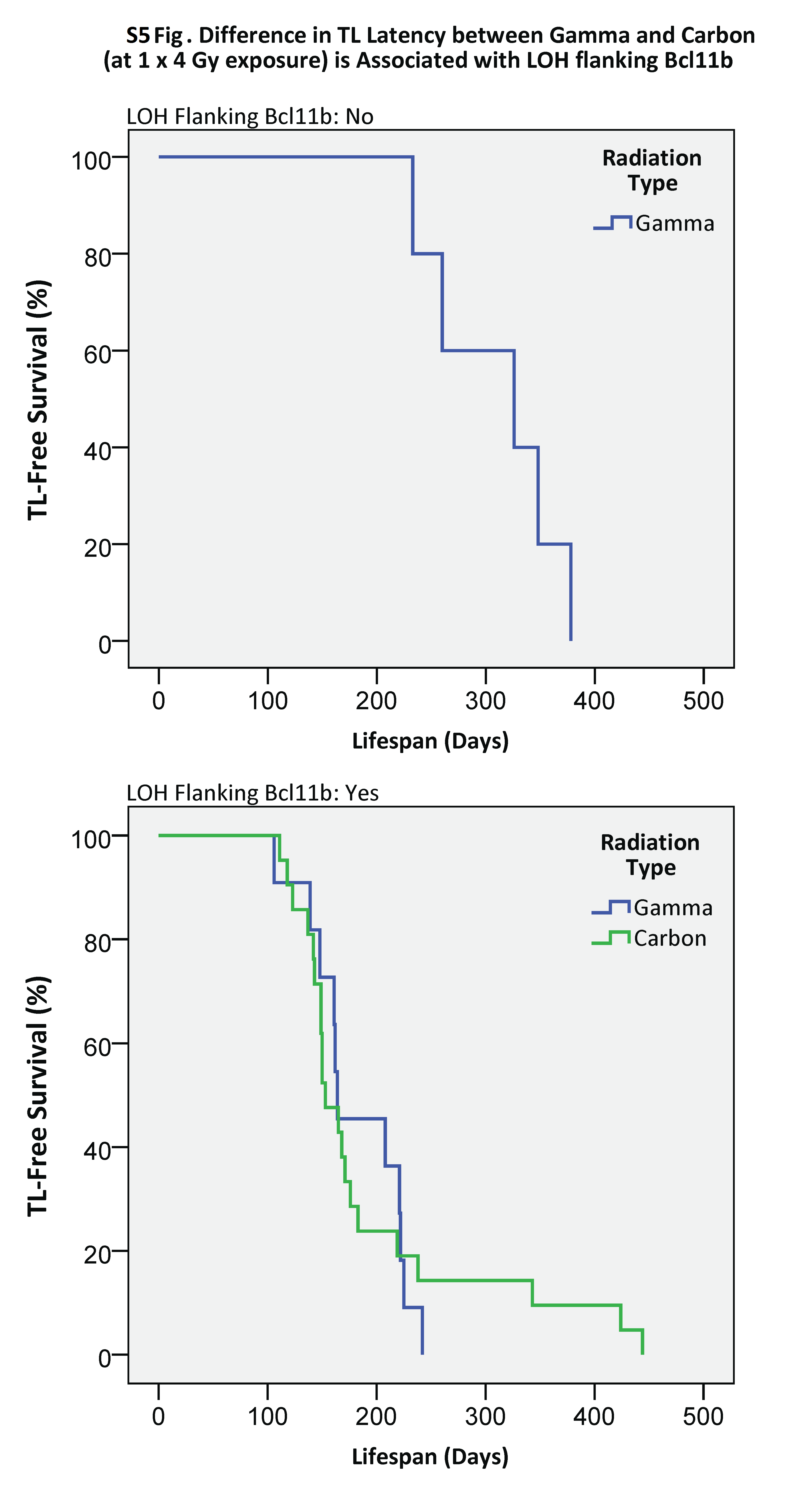

Supplement: S5 Fig — When the mice without LOH flanking Bcl11b are separated (n = 5), the remaining TL in mice treated with 1 x 4 Gy show no difference in latency between gamma ray (n = 11) or carbon ion irradiation (n = 21, P = 0.96, Log-Rank). (TIF) [file pone.0130666.s007.tif]

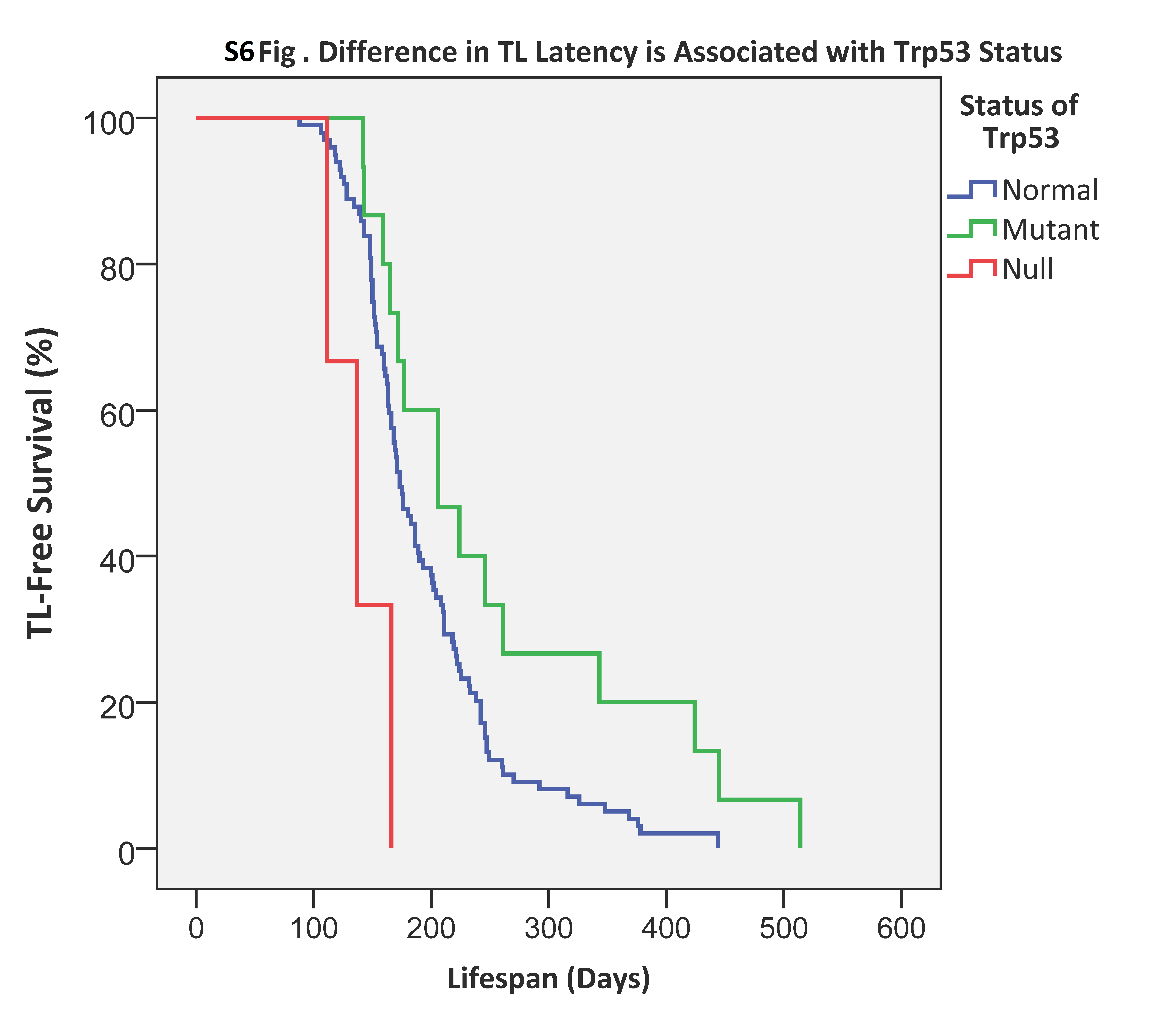

Supplement: S6 Fig — Across the whole TL cohort, TL with mutant Trp53 expression (n = 15) showed an increased tumour latency (P = 0.018, Log-Rank), while TL with null Trp53 expression (n = 3) showed decreased tumour latency (P = 0.09, Log-Rank) compared to Trp53 wildtype TL (n = 99). This effect is likely a distinction between rare, early Trp53 loss events which were produced directly by the radiation which lead to rapid tumour progression, the majority of tumours which have no selective pressure to acquire Trp53 mutations due to alternate events which bypass Trp53, and more indolent tumours which acquire Trp53 mutations late due to sustained selection pressure. (TIF) [file pone.0130666.s008.tif]

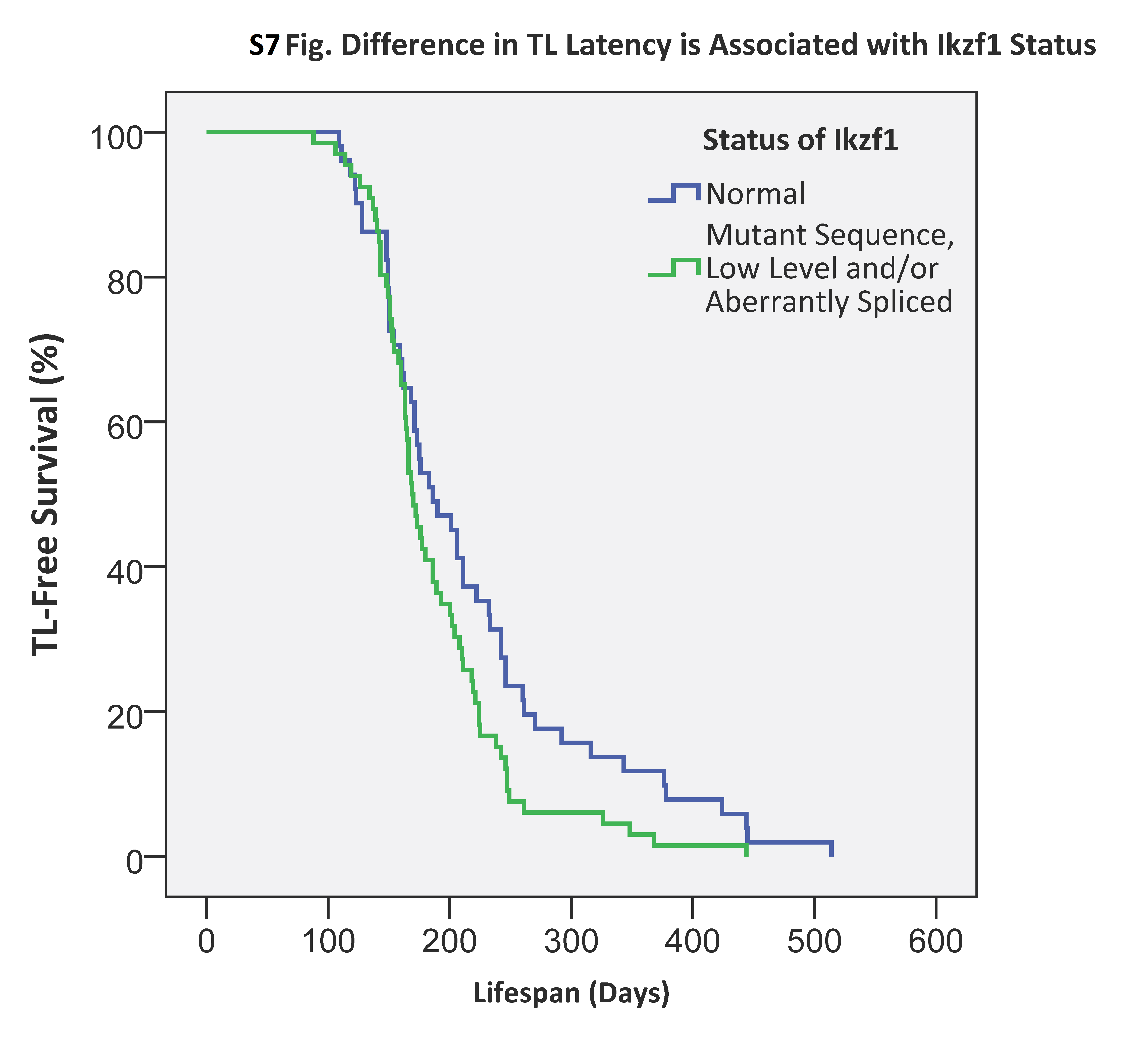

Supplement: S7 Fig — Across the whole TL cohort, TL with normal Ikzf1 expression (n = 55) had significantly increased tumour latency than TL with mutant sequence (n = 66), low levels and/or aberrant splicing (P = 0.037, Log-Rank). (TIF) [file pone.0130666.s009.tif]
