# Supplementary material for: Genetic Analysis of T Cell Lymphomas in Carbon Ion-Irradiated Mice Reveals Frequent Interstitial Chromosome Deletions: Implications for Second Cancer Induction in Normal Tissues during Carbon Ion Radiotherapy
Source: PLoS One. 2015 Jun 30;10(6):e0130666. doi: 10.1371/journal.pone.0130666 (PMC4488329; doi:10.1371/journal.pone.0130666)
Supplement: S1 Methods — (PDF) [file pone.0130666.s010.pdf]

Cohort Selection and Sample Processing
For each mouse, the enlarged thymus was removed, trimmed of connective tissue, weighed, and then divided into several parts for long-term storage. One portion of the enlarged thymus tissue was used to prepare a single cell suspension in phosphate buffered saline (PBS) on ice by grinding with a glass pestle over sterile wire gauze and filtering through fresh wire gauze. The cells were washed in PBS and pelleted by gentle centrifugation (4°C, 300 × *g*, 5 minutes). Some of the cells were resuspended in PBS and frozen at -80°C for later DNA extraction. Some of the cells were lysed and homogenised by vortexing in guanidinium isothiocyanate (GTC) buffer before freezing at -80°C for later RNA extraction. In some cases the pelleted cells were resuspended in ice cold PBS plus 1% fetal calf serum (PBS+FCS) and the viable cell concentration was determined by haemocytomer counts for immediate immunophenotyping by flow cytometry.

Nucleic Acid Extraction
Frozen cell suspensions in PBS were thawed briefly, and an aliquot taken for extraction using the Maxwell® 16 Tissue DNA Purification Kit and the Maxwell® 16 Research Instrument. After automated DNA extraction, the resulting DNA in solution was precipitated with ethanol, washed and resuspended in water for long-term storage at -20°C. For isolating RNA the cell suspensions in GTC buffer were thawed briefly, and again an aliquot was taken for pre-treatment to flocculate genomic DNA before adding the cleared lysate to the Maxwell® 16 Total RNA Purification Kit. After automated RNA extraction, the resulting RNA in solution was precipitated with ethanol, washed and resuspended in water for long-term storage at -20°C. Both DNA and RNA concentrations were determined by A260/280 spectrophotometry using a Nanodrop-1000 (Thermo Scientific). For each tumour sample, cDNA was reverse transcribed from 5 µg of total RNA, using a modified M-MLV reverse transcriptase and random hexamer oligonucleotides. To test success of RNA extraction and cDNA preparation, primers to amplify a product (approx. 700 bp) from the abundant *Gapdh* transcript were used to ensure amplification from all samples.

Immunophenotyping by Flow Cytometry
For flow cytometry analysis, 5 × 10^5 cells from the single cell suspension prepared at necropsy were incubated with antibodies as shown below in a total staining volume of 35 µL PBS+1% FCS for 30 minutes on ice, protected from light. The labelled cells were washed with fresh PBS+FCS and then pelleted by centrifugation at 4°C, 300 × *g*, 3 minutes. The labelled cells were resuspended in 400 µL PBS+FBS and analysed by Guava EasyCyte Plus instrument (Millipore), measuring forward-and side-scattered light, as well as FITC and PE fluorescence signals. Instead of relying only on generic isotype-matched antibody controls, normal unirradiated thymus cells were routinely analysed in parallel which allowed the delineation of negative- and positive-stained populations based on the normal presence of these cell populations.

Reaction	Antibody 1	Antibody 1 Volume	Antibody 2	Antibody 2 Volume
1	Rat anti-mouse CD8a – FITC<br>Monoclonal (Clone: 53-6.7)<br>(eBioscience Cat# 11-0081-81,<br>RRID:AB_464914)	5 µL (25 µg/mL)	Rat anti-mouse CD4 – PE<br>Monoclonal (Clone: RM4-5)<br>(eBioscience Cat# 12-0042-82, RRID:AB_465510)	5 µL (20 µg/mL)
2	Rat anti-mouse CD90.2 – FITC<br>Monoclonal (Clone: 30-H12)<br>(BD Biosciences Cat# 553012,<br>RRID:AB_394550)	5 µL (5 µg/mL)	Rat anti-human/mouse CD45R – PE<br>Monoclonal (Clone: RA3-6B2)<br>(eBioscience Cat# 12-0452-82, RRID:AB_465671)	5 µL (10 µg/mL)

The RRID is a permanent unique identifier of these antibodies and can be used at http://antibodyregistry.org/

CD4+ CD8+ Phenotype

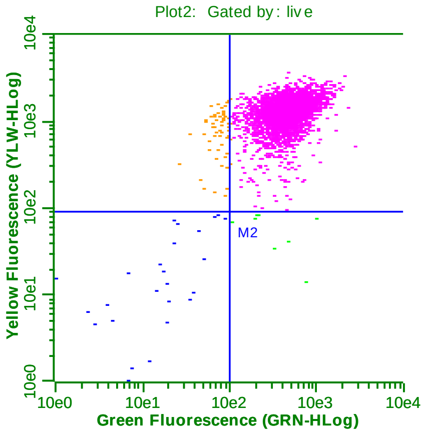

Tumour ID: 23305

CD4+ CD8- Phenotype

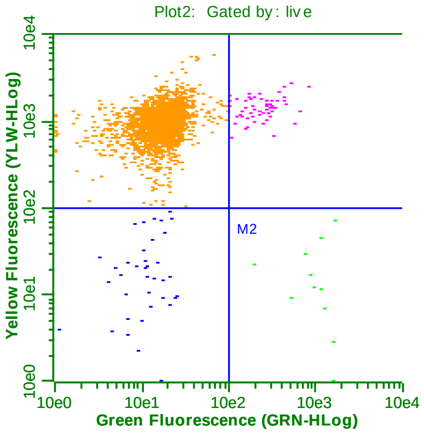

Tumour ID: 23713

CD4- CD8+ Phenotype

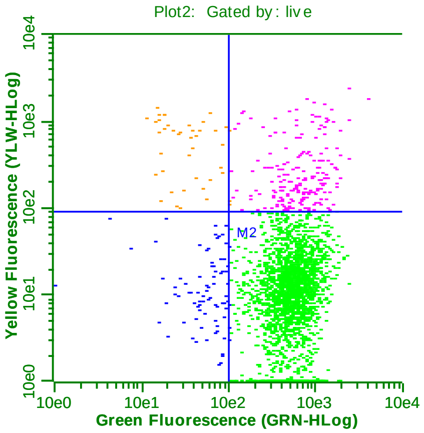

Tumour ID: 23547

CD4- CD8- Phenotype

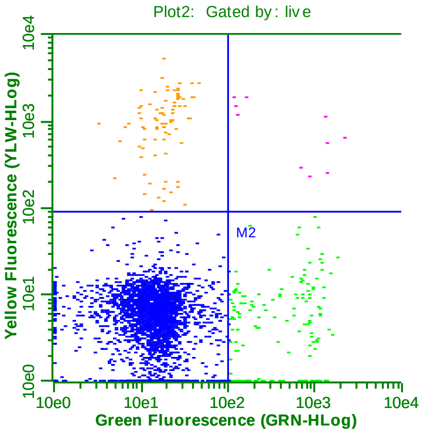

Tumour ID: 23990

CD4+/- CD8+/- Phenotype

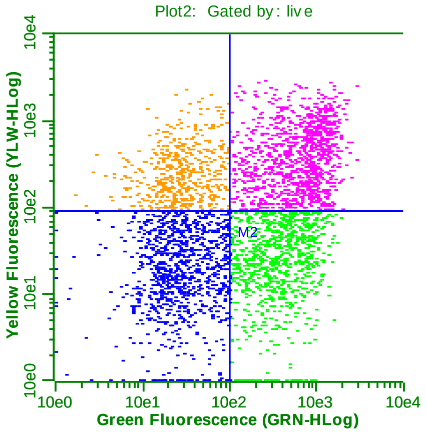

Tumour ID: 23329

CD4+ CD8+/- Phenotype

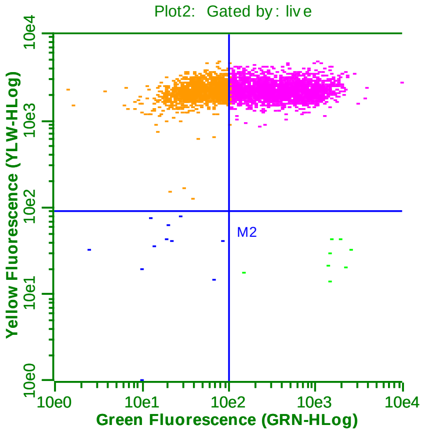

Tumour ID: 23356

CD+/- CD8+ Phenotype

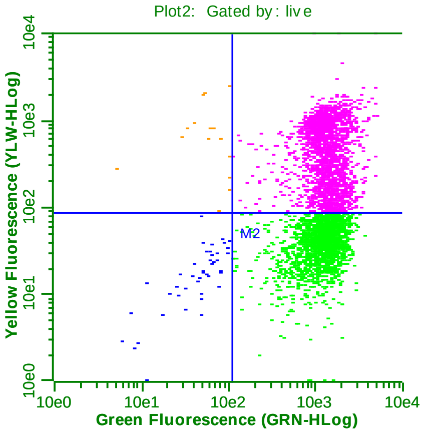

Tumour ID: 23374

Mixed Phenotype

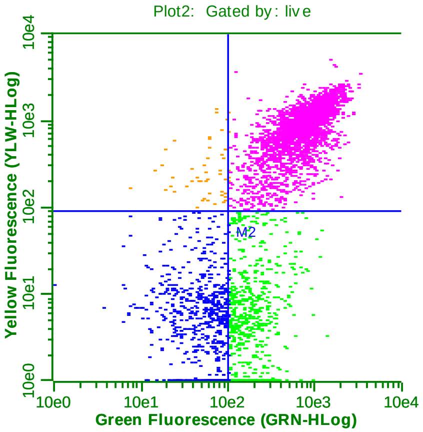

Tumour ID: 23780

Data representative of the eight observed CD4/CD8 immunophenotypes for carbon ion-induced TLs are shown. CD8-FITC signal is shown by Green Fluorescence on the *x*-axis, while the CD4-PE signal is shown by the Yellow Fluorescence on the *y*-axis.

Locations Interrogated for Loss of Heterozygosity and PCR and Analysis Conditions

Chromosome	Location	Distance from Target[a]	Locus	B6 Product Size	C3H Product Size	PCR Annealing Temp	PCR Cycles	Analysis Method	Forward Primer Sequence 5` - 3`	Reverse Primer Sequence 5` - 3`
11	6.9	-4.8	D11Mit71	214	238	58	30	Capillary Electrophoresis	GCCATACCTGGTAGCGTGTT	AATTTTCAGATGTAGCCATAAGCC
11	9	-2.7	D11Mit62	148	160	57	35	Capillary Electrophoresis	GAATAACCCATGTTTATATCGGTG	CTCTGGACTTGTGTTCTATGCC
11	12.3	0.6	D11Mit2	122	137	55	32	Melt Curve Analysis	TCCCAGAGGTCTCCAAGACA	CCACAGTGTGTGATGTCTTC
11	17.8	6.1	D11Mit77	152	162	58	30	Capillary Electrophoresis	GTATTCAAATGACTTCTGCCTGG	TTGAAATGGTCTTCAAGTGGC
11	44.7	33	D11Mit20	116	150	58	30	Capillary Electrophoresis	CCTGTCCAGGTTTGAGAGGA	CTTGGGAGCCTCTTCGGT
11	69.8	58.1	Acrb	160	170	58	30	Capillary Electrophoresis	GCTGCTGAACAAGTAGGGTC	CCGTCATTGCTAAAGGGAAG
11	98.7	87	D11Mit14	155	164	58	30	Capillary Electrophoresis	CCACTTAGTATATCTTGTCC	GCATGACTTGGCCTATCACC
11	116.4	104.7	D11Mit203	141	131	58	30	Capillary Electrophoresis	GACAGCACTGAAAGTCTGATGC	AGTCATGTTCTAATTGTGCTTACACA
12	5.4	-102.6	D12Mit37	140	118	58	30	Capillary Electrophoresis	AAGTTTGAACACAGAACACTCAGC	TCTGGTTTGCAGGAAGCC
12	10.9	-97.1	D12Mit182	132	132	58	30	Capillary Electrophoresis	GTACATACAATACATCACACAAACGG	GGCAAGAAAACAGACCAATAGG
12	25.9	-82.1	D12Mit105	140	108	58	30	Capillary Electrophoresis	TACACACATACACATGCTCATATGC	TGTCCCTATAGAGAACCCTAATGC
12	77.9	-30.1	D12Mit52	140	118	58	30	Capillary Electrophoresis	CCATCTTCTGGCATTTTGCT	AGACAGGAGGGTCCCAAAGT
12	82.1	-25.9	D12Mit14	130	146	58	30	Capillary Electrophoresis	GAGAGAGTTTCCCTTGTTGTCG	AACTCTCTAGGCAGAGTGCCC
12	102.9	-5.1	D12Mit101	170	118	58	30	Capillary Electrophoresis	GCTTTTCCTTATCAAGATATGCG	GCAGCAGAAAGAGAGGGAAA
12	107.4	-0.6	D12Mit132	131	112	59	33	Melt Curve Analysis	CCATATACATTTCTAACACCCTTGC	AGAACTTACTTCTAGTGGAGACAATGC
12	113.9	5.9	D12nds2	195	178	55	32	Capillary Electrophoresis	ACATGGTAATTTATGGGCAA	CTGGATACCTGCAATAGTAGA
19	3.6	-29.2	D19Mit68	136	122	58	30	Capillary Electrophoresis	CCAATACAAATCAGACTCAATAGTCG	AGGGTCTCCCCATCTTCCTA
19	5.3	-27.5	D19Mit59	199	139	58	30	Capillary Electrophoresis	CTCTAACTATCCTCTGACCTTCACA	TTTTAAGCAGAACATTGAGGACC
19	18.7	-14.1	D19Mit41	160	174	58	30	Capillary Electrophoresis	AGCCCTCCACCCAGTTTC	TCTGGGGAAAAAGGATGAGA
19	32.8	0	Pten	242	≈250[b]	60	30	4% Agarose Gel Electrophoresis	TGCATGTATGTTTTTGTCTTGG	TTCGACCCTACAAGAATCCAGA
19	39.5	6.7	D19Mit19	142	116	55	36	4% Agarose Gel Electrophoresis	CCTGTGTCCATACAGGCTCA	ACCATATCAGGAAGCACCATG
19	45.2	12.4	D19Mit53	110	102	58	30	Capillary Electrophoresis	GCACGCCACAACTCAGAG	AGAAAAGGTTCTCCTACCTCTCG
19	47.9	15.1	D19Mit91	112	90	58	30	Capillary Electrophoresis	GGGTTGGCTCAATACTCCAA	CCCCCACCTGGTATCTTGAG

Location and Distance from Target are given in Mb (negative and positive distances are centromeric and telomeric to target, respectively) using GRCm38 positioning.
[a] Targets are *Ikzf1*, *Bcl11b* and *Pten* on chromosomes 11, 12 and 19 respectively.
[b] Size is estimated.

- **Capillary Electrophoresis** was conducted by applying the PCR products onto an *eGene HAD-GT12 Capillary Electrophoresis* instrument and separating using a *QIAxcel DNA High Resolution kit* along with 15 bp and 500 bp alignment markers. LOH was determined by the presence or absence of the peaks corresponding to those produced by the C57BL/6 and C3H control DNA samples.
- **Melt Curve Analysis** was performed by adding 1 µL of EvaGreen (20X) dye to the 25 µL reactions after the completion of the PCR and reading the samples during a melt-curve analysis on the Mx3000P real-time PCR system (Agilent). LOH was determined by the presence or absence of the peaks corresponding to those produced by the C57BL/6 and C3H control DNA samples.
- **Agarose Gel Electrophoresis** was performed by separation of PCR products in a 4% NuSieve Agarose (Lonza) gel at 100V for 30 minutes. LOH was determined by the presence or absence of the bands corresponding to those observed in the C57BL/6 and C3H control DNA reactions after staining with ethidium bromide and visualisation under UV light.

Sequencing Analyses and PCR Conditions

PCR was performed as follows:
Total Reaction Volume = 25 µL : 0.5 units Takara rTaq, 1X rTaq PCR Buffer, 2.5 mM $MgCl_2$, 0.25 mM each dNTP, 10 ng each primer (see table), TL cDNA (equivalent to 5 ng total RNA)
Reactions were prepared on ice, and run using a 'hot-start' protocol (samples were loaded once the block temperature exceeded the primer annealing temperature) on a Veriti Thermal Cycler (Applied Biosystems).

Locus	Fragment	Coverage	PCR Annealing Temp	Cycles	Forward Primer Sequence 5` - 3`	Reverse Primer Sequence 5` - 3`	Primer References	Reference Sequence for Mutation Detection
Notch1	HD Domain	Exon 26-27	60	32	AACAGTGCCGAATGTGAGTG	CACAAAGAACAGGAGCACGA	10.1182/blood-2011-01-327619	NM_008714.3
	PEST Domain	Exon 34 aa. 2238 ->	60	32	AGTCACCCCATGGCTACTTG	CCTGAAGCACTGGAAAGGAC	10.1182/blood-2011-01-327619	
Pten	1	Exon 1-9	61	45	CAGCAGCTTCTGCCATCT	TGCAATCTGACACAATGTCCTA	10.1016/j.mrfmmm.2009.12.011	NM_008960.2
		Alternate reverse sequencing primer				TGTGAATGCTGATCTTCATC		
Trp53	1	Exon 1-11	61	40	AGTTCTGTAGCTTCAGTTCATTGGGACCATCCTG	CCAGCAGAGACCTGACAACTAT	In-house design	NM_011640.3
	1	*Additional internal sequencing primers*			TCTGCCTGTCTTCCAGATACT	CTTATCCGGGTGGAAGGAAAT		
Ikzf1	1	Exon 1-4	60	40	CAATGGATGTCGATGAGGGTC	GGTTGCACTGGAAAGGCCGT	http://www.jstor.org/stable/3580875	NM_001025597.1
	2	Exon 3-6	60	40	AGGCATTAGACTTCCTAACG	TCTGAGGCATAGAGCTCTTAC	http://www.jstor.org/stable/3580875	
	3	Exon 6-7	60	40	CAAGATAGGAGCAGAGAGGTC	CGCAAATCAAACGCCAAACAAC	http://www.jstor.org/stable/3580875	
	4	Exon 2-7	64	30	CACTACCTCTGGAGCACAGCAGAA	CATAGGGCATGTCTGCACGGCACT	http://www.jstor.org/stable/3580875	

Ikzf1 Transcript Analysis

In the case of *Ikzf1* (which normally produces multiple PCR bands corresponding to the several splicing isoforms), the band corresponding to the full-length isoform was excised and sequenced (as well as any novel bands). To reduce the chance of false-negatives from failure to detect mutations which might result in non-amplification of the *Ikzf1* transcript from the standard primer sets, multiple real-time quantitative PCRs were conducted on the tumour cDNA of any tumours with absent, low or uneven amplification of the full-length transcript or the various overlapping fragments. These PCRs included primers spanning each of the exon-exon junctions which enabled the identification of missing junctions, 5' or 3' truncated transcripts and normal transcripts expressed at low/very low levels. Tumour cDNA was amplified in real-time by RT-PCR in 10 µL duplicate reactions [Takara Premix ExTaq (ROX Plus), 1X EvaGreen Dye, cDNA, 0.33 µM each primer] for *Gapdh* (normalising control) and 9 in-house designed primer pairs covering *Ikzf1* exon-exon boundaries (Exon 0:1, Exon 1:2, Exon 2:3, Exon 2:4, Exon 3:4, Exon 3:5, Exon 4:5, Exon 5:6, Exon 6:7) as shown below. Reactions underwent initial denaturation (95°C, 20 s) and were then amplified for 35 cycles (95°C, 5 s; 60°C, 30 s) measuring EvaGreen and ROX fluorescence at the end of each annealing/extension phase.

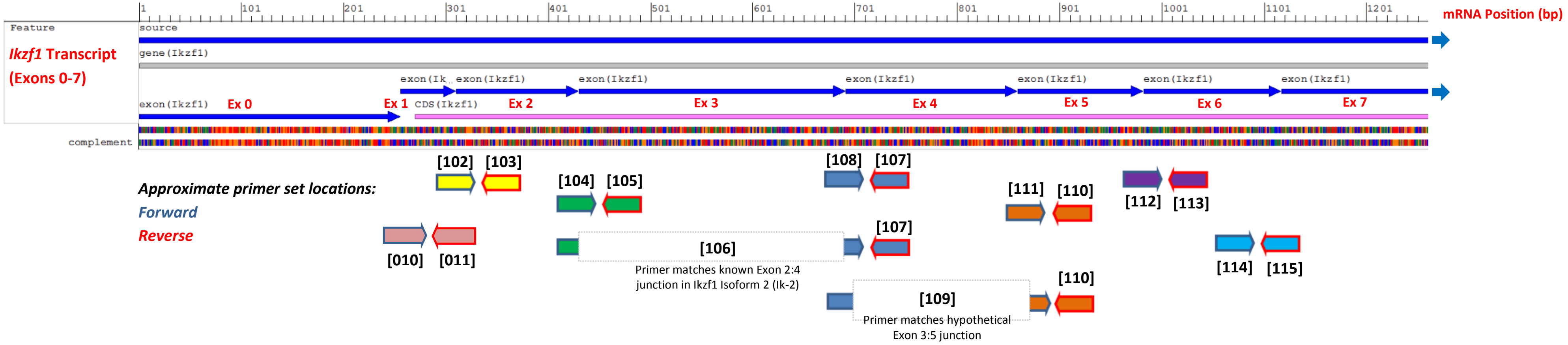

Set	Primer 1 Name	Primer 1 Sequence (5'-3')	Primer 2 Name	Primer 2 Sequence (5'-3')
cDNA Normalisation	Gapdh-RQ-F	GTCAGCAATGCATCCTGCA	Gapdh-RQ-R	GTGGTCATGAGCCCTTCCA
Exon 0:1 Boundary to Exon 1:2 Boundary	Ikzf1-RQ-F[010]	GGCGCACAAATCCACATAAC	Ikzf1-RQ-R[011]	AAGTTTCAGGAAAGGAGAGCC
Exon 1:2 Boundary to Exon 2	Ikzf1-Ex1-2-F [102]	GTCCCAAGTTTCAGGAAAGGA	Ikzf1-Ex2-R [103]	ACTCTTGGAGTTCTGCTGTG
Exon 2:3 Boundary to Exon 3	Ikzf1-Ex2-3-F [104]	CGAGGCATGGCCAGTAAT	Ikzf1-Ex3-R [105]	TCGTAAATCCTCTGCACATTCT
Exon 2:4 Boundary to Exon 4 (Detects skipping of Exon 3)	Ikzf1-Ex2-4-F [106]	GATCGAGGCATGGGTGAAC	Ikzf1-Ex4-R [107]	CCGAGTGCAGCTTGATGT
Exon 3:4 Boundary to Exon 4	Ikzf1-Ex3-4-F [108]	AAGAAGTCATACTGGTGAACGG	Ikzf1-Ex4-R [107]	CCGAGTGCAGCTTGATGT
Exon 3:5 Boundary to Exon 5 (Detects skipping of Exon 4)	Ikzf1-Ex3-5-F [109]	AGAAGTCATACTGTTGGTAAGCC	Ikzf1-Ex5-R [110]	TAGTTGTGGCATCGCTCTTTAT
Exon 4:5 Boundary to Exon 5	Ikzf1-Ex4-5-F [111]	GACGCACTCCGTTGGTAAG	Ikzf1-Ex5-R [110]	TAGTTGTGGCATCGCTCTTTAT
Exon 5:6 Boundary to Exon 6	Ikzf1-Ex5-6-F [112]	GGGCATGTACCCAGTCATTAAG	Ikzf1-Ex6-R [113]	GGACCTCTCTGCTCCTATCTT
Exon 6 to Exon 6:7 Boundary	Ikzf1-Ex6-F [114]	CTGTGCAAGATAGGAGCAGAG	Ikzf1-Ex6-7-R [115]	AGGCACTTGTCTCCAAGAAAT

The bar chart at right shows the results of four representative samples:

(Mean Cq of Each Product – Mean Cq of Gapdh Control) = Cq^N *Graph axis shows: Cq^N of Normal Thymus cDNA – Cq^N of Tumour*

(**S22691**) The expression of the first two products are normal, but with almost no expression of all products from the exon 2:3 boundary onwards, indicating the expression of a truncated transcript. (**S22708**) The expression of all products was within 1 cycle of normal, except for the presence of an exon 3:5 boundary (red bar, deletion of exon 4). (**S22539**) Expression of all products was evenly and consistently depressed indicating down-regulated but otherwise normal expression. (**S22533**) Expression of all products was evenly and consistently depressed, except for the exon 5:6 junction which was almost absent, which was revealed by sequencing to be caused by irregular splicing resulting in a novel exon between exons 5 and 6.

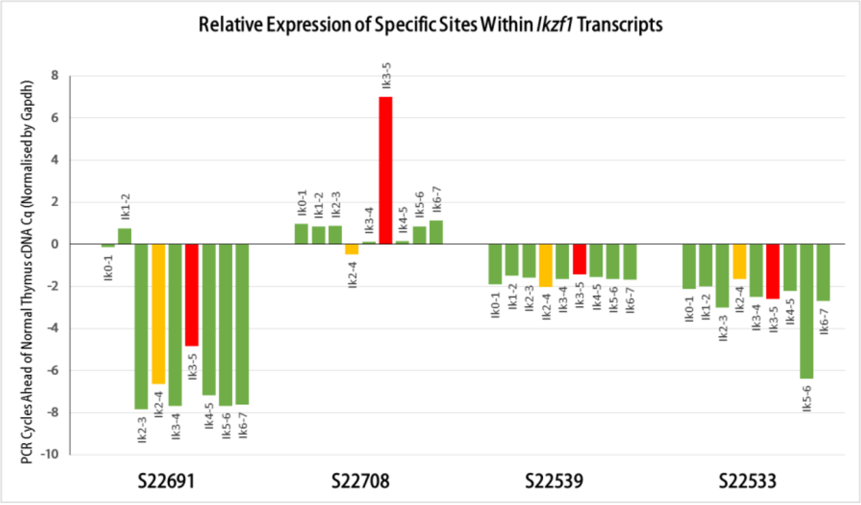

DNA Copy Number Analysis at Sites in and Flanking *Bcl11b*

Tumour DNA was amplified by PCR in triplicate 10 µL reactions:
Premix ExTaq with ROX (1×) [Takara Biotech]
EvaGreen dye (1×) [Biotium], 50 ng DNA and 0.33 µM of each primer.
The reactions were conducted for each sample using each of four primer sets as shown in the table.

Set	Primer 1 Name	Primer 1 Sequence (5'-3')	Primer 2 Name	Primer 2 Sequence (5'-3')
Tert DNA Copy Number Reference	Tert-CN1-F [062]	AGAGAGCACATTCCAGAAGAAC	Tert-CN1-R [064]	GCACACAGGAGACACTGATAC
Bcl11b DNA Copy Number Test	Bcl11b-CN2-F [085]	AACCTCTCCTTCCCTTCTCT	Bcl11b-CN2-R [087]	CTGGACTCTTCAGCTTCCATAC
D12Mit132 DNA Copy Number Test	12-132-CN1-F [088]	TTGGATTTCTAGCGGGATTATGA	12-132-CN1-R [089]	TCCAGGAAATTGCTCATCTCTAC
D12nds2 DNA Copy Number Test	12-nds2-CN1-F [090]	CACACACACACAGAGGAACT	12-nds2-CN1-R [091]	ACCACTCAGTCCTGTCACTA

After an initial denaturation for 20 s at 95°C, the PCR was cycled 35 times (95°C, 5 s; 60°C, 30 s) reading EvaGreen and ROX fluorescence at the end of each cycle (MX3000P, Agilent). The PCR was followed by melt curve analysis to confirm the amplification of the expected amplicons. For each sample, the mean C_q of the triplicate reactions was averaged (from the ROX normalised data, using a single threshold for all four primer sets, for every PCR run) and the value for the Tert DNA Copy Number reference reaction was subtracted from the value of each of the three test reactions.

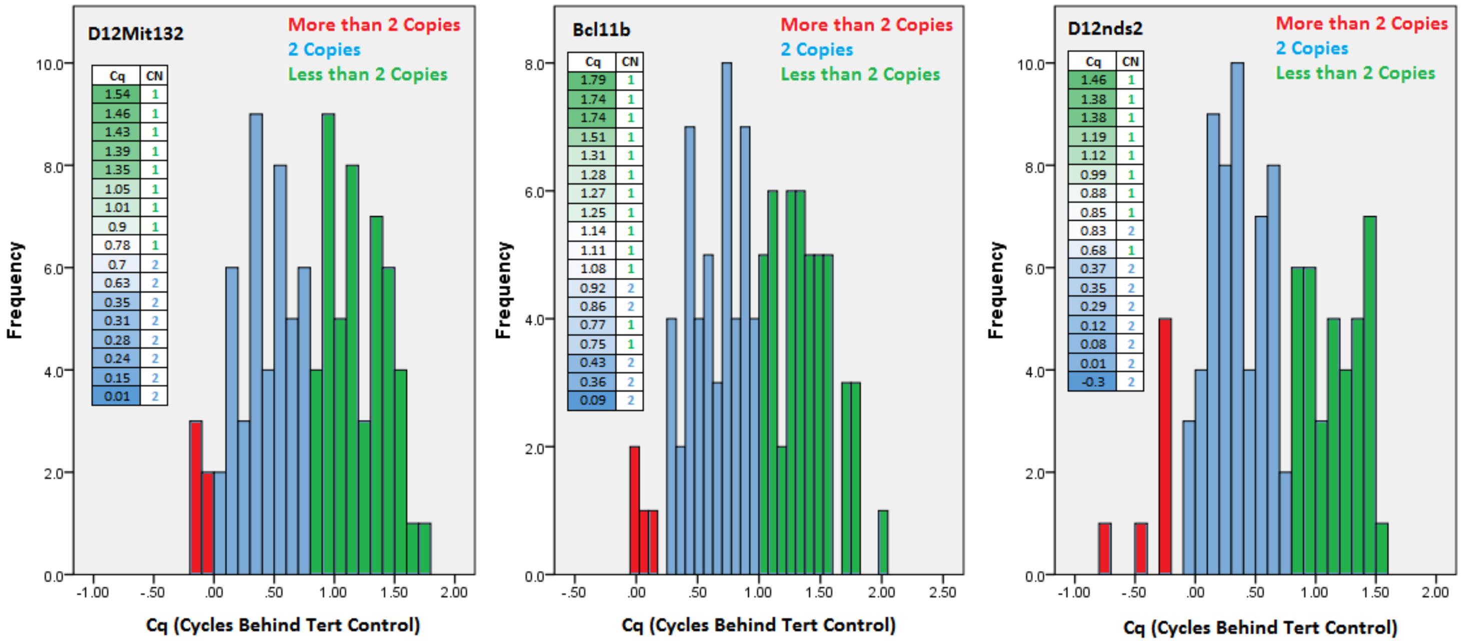

The results for the whole cohort are shown for each of the three test sites as histograms for the Tert-normalised Cq. Using samples for which the DNA Copy Number PCR results and CGH results were both available (listed in tables within each panel, Cq: Tert-normalised Cq, CN: Nominal DNA copy number classified by CGH), cut-off Cq values were derived to divide samples into '2 copies' or 'less than 2 copies' groups. Applying these cut-offs to the whole cohort (colour-coding of histograms) confirmed that these thresholds naturally separated the two peaks representing cells with one or two copies of DNA at the site in question.

Variation from 'perfect' values can be attributed to both experimental variation, and heterogeneity within the tumour samples (not all cells will necessarily carry the same number of copies). The DNA copy number result by CGH is similarly based on a threshold classification of normalised Test : Reference probe signal ratios.

With the combined LOH results from the upstream D12Mit132 and downstream D12nds2 sites and the DNA copy number results as derived above, the samples were further classified into one of seven groups as shown below.

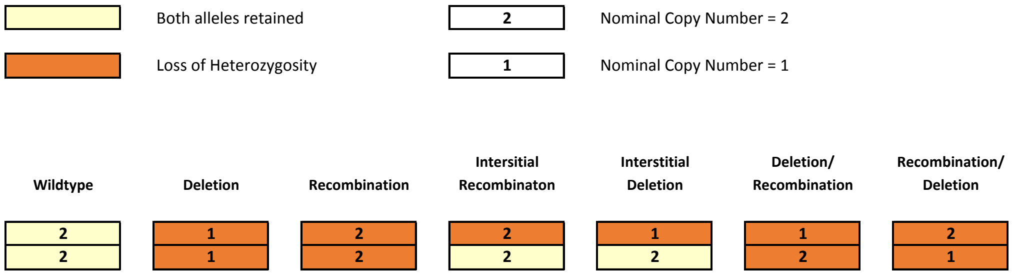

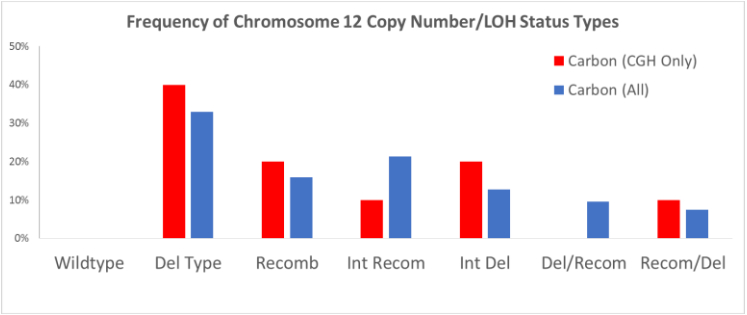

Comparison of the Chromosome 12 Copy Number/LOH Status Types between the initial analysis using only the carbon TL with both CGH and LOH data (red bars) and the extension of the analysis to all carbon TL using the DNA Copy Number PCR and LOH data (blue bars) shows that the pattern observed in the sub-cohort was upheld across the whole cohort.

Validation of *Ikzf1* and *Pten* Mutations

The *Ikzf1* and *Pten* genes contain several homopolymer tracts of 6 or more identical nucleotides in the coding sequence which are known to be sensitive to errors during replication, producing mutation hot-spots. However, to demonstrate that tumours exhibiting such mutations in the cDNA sequencing results were the result of *bone fide* DNA mutations rather than errors introduced during reverse transcription or PCR amplification, exon 4 and exon 7 from *Ikzf1*, and exons 5, 6 and 8 from *Pten* were amplified from tumour DNA of samples (*n*=56) showing the corresponding mutations (together these exons represented all of the homopolymer tract insertion/deletions and most of the mutations overall). Exons were amplified from tumour DNA for 35 cycles (95°C, 5s; 60°C, 20s; 72°C, 20s) in 25 µL reactions (20 ng DNA, 0.4 µM each primer). PCR amplicons were excised after separation by agarose gel electrophoresis and sequenced with both forward and reverse PCR primers, comparing the results against the reference sequences (*Ikzf1*: NC_000077.6, 11686213…11772926) (*Pten*: NC_000085.6, 32757577…32826160). In each case, the mutations observed in the cDNA were reflected in the DNA sequence.

Primer Set	Forward Primer Name	Forward Primer Sequence	Reverse Primer Name	Reverse Primer Sequence
Ikzf1 Exon 4	Ikzf1-X4-Seq-F [098]	CGCTCTCTCTCAGTGCTTAC	Ikzf1-X4-Seq-R [099]	GGAATAAGGGATTACCACATAGGA
Ikzf1 Exon 7	Ikzf1-X7-Seq-F [100]	CAGTGCCAACTATGAGAAGGAG	Ikzf1-X7-Seq-R [101]	ACCAGCTATCTTTGTGCTTCA
Pten Exon 5	Pten-X5-Seq-F [126]	TGAAGACCATAACCCACCAC	Pten-X5-Seq-R [127]	ATGACCTTATGTTGGGGCAC
Pten Exon 6	Pten-X6-Seq-F [128]	CTGCAGTACAGAGACCATTG	Pten-X6-Seq-R [129]	TGATGAGGACCTGGTTGTA
Pten Exon 8	Pten-X8-Seq-F [124]	CACAAGGTGTTTGCCTTC	Pten-X8-Seq-R [125]	GCTCTTAGCTTCACAATCAG

Primers were all designed in-house

Representative Results of DNA Sequence from *Ikzf1* Exon 7 for Four TL Compared to Germline Sequence

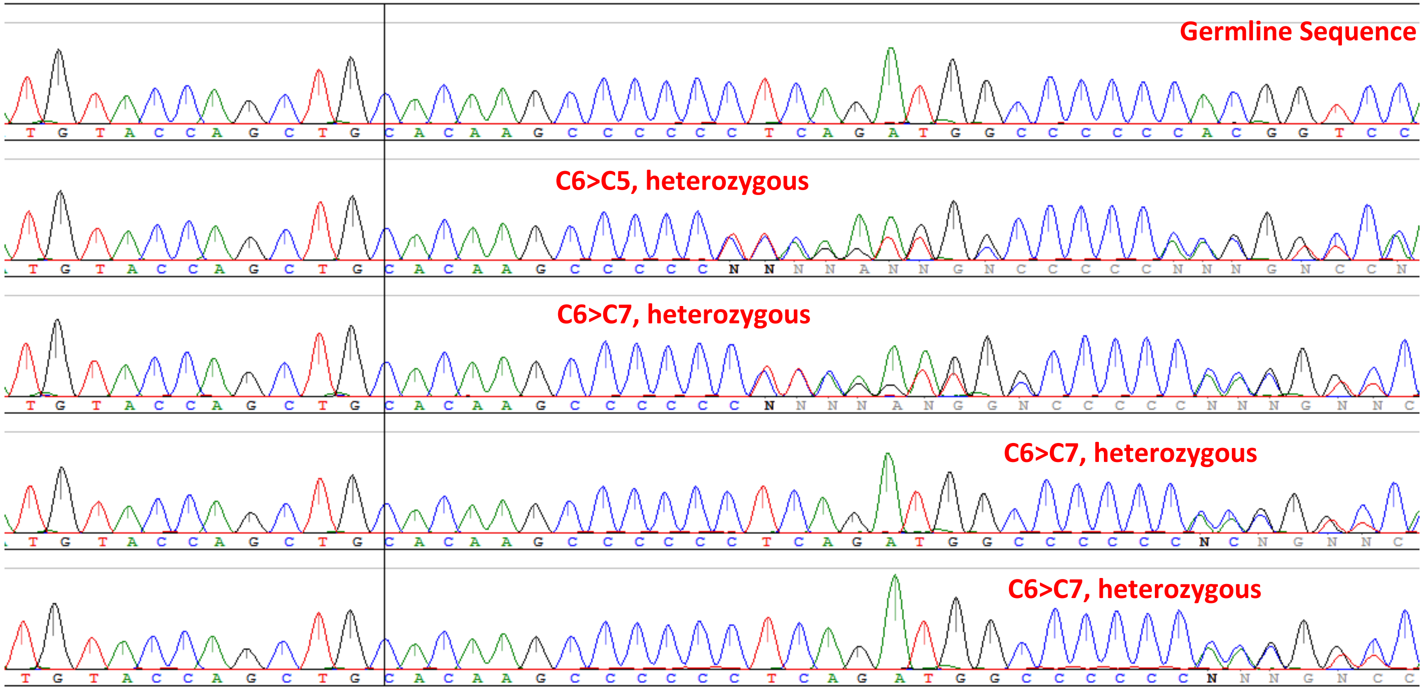

Notch1 5' Deletion PCR
Original Method Sourced From:
Oncogenic activation of the Notch1 gene by deletion of its promoter in Ikaros-deficient T-ALL, Jeannet et al. 2010 (http://bloodjournal.hematologylibrary.org/content/suppl/2010/08/27/blood-2010-05-286658.DC1/Document1.pdf)

PCR was performed as follows:
Total Reaction Volume = 25 µL : 0.5 units Takara rTaq, 1X rTaq PCR Buffer, 2.5 mM $MgCl_2$, 0.25 mM each dNTP, 10 ng each primer (Notch1 5' Deletion pair or *Tert* Control pair), 20 ng TL DNA

Notch1 5' Deletion PCR Primers
Forward: 5'-ATGGTGGAATGCCTACTTTGTA-3' (nucleotide -8388 to -8366 from exon 1)
Reverse: 5'-CGTTTGGGTAGAAGAGATGCTTTAC-3' (reverse, nucleotide 3929 to 3942).

Tert DNA Amplification Control Primers
Forward: 5'-AGAGAGCACATTCCAGAAGAAC-3'
Reverse: 5'-GCACACAGGAGACACTGATAC-3'

Thermal-cycler Conditions
Hot Start, 94°C 5 min, 35 cycles (94°C 30s, 58°C 30s, 72°C 45s)

Amplicons were separated by 1.5% agarose gel in 0.5X TBE, run at 100V for 30 minutes, and visualised by ethidium bromide staining and digital imaging under UV light. Figure shows a gel photograph of two control samples (Deletion negative, lane 1; Deletion positive, lane 2) and 11 TL samples (1 per lane) with Notch1 5' Deletion products in the top row, and Tert DNA amplification controls in the second row. Tumours positive for the specific deletion are in lanes 10 and 12, while all tumours show normal amplification of a product from a normal 2-copy gene (Tert). The lower rows of the gel (which contain different samples) have been cropped out for clarity.

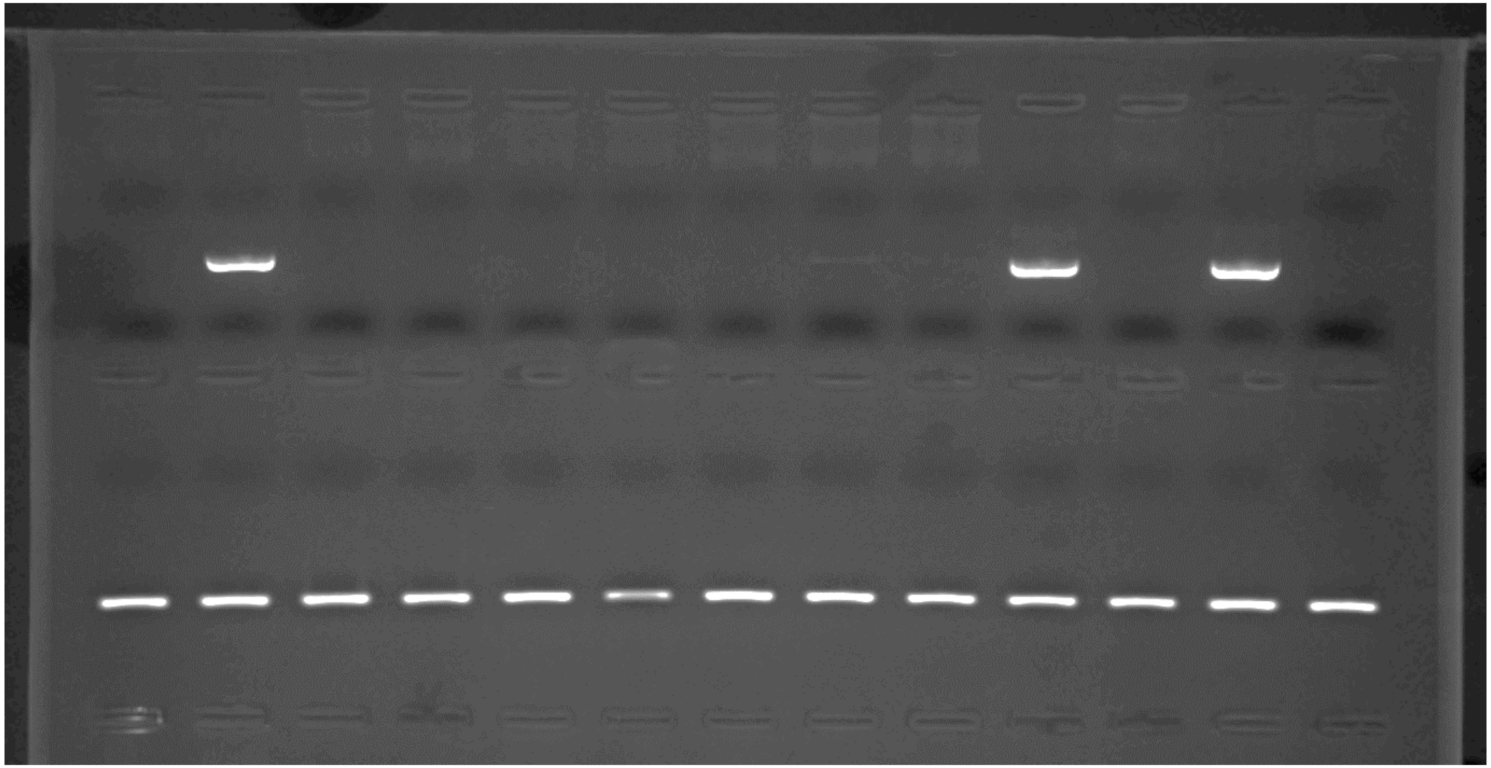

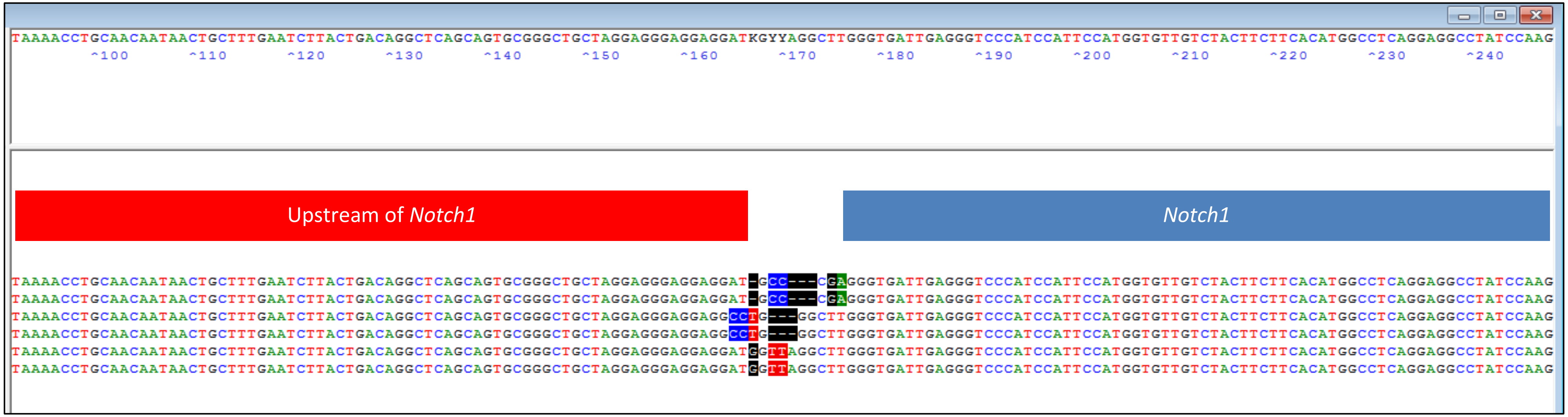

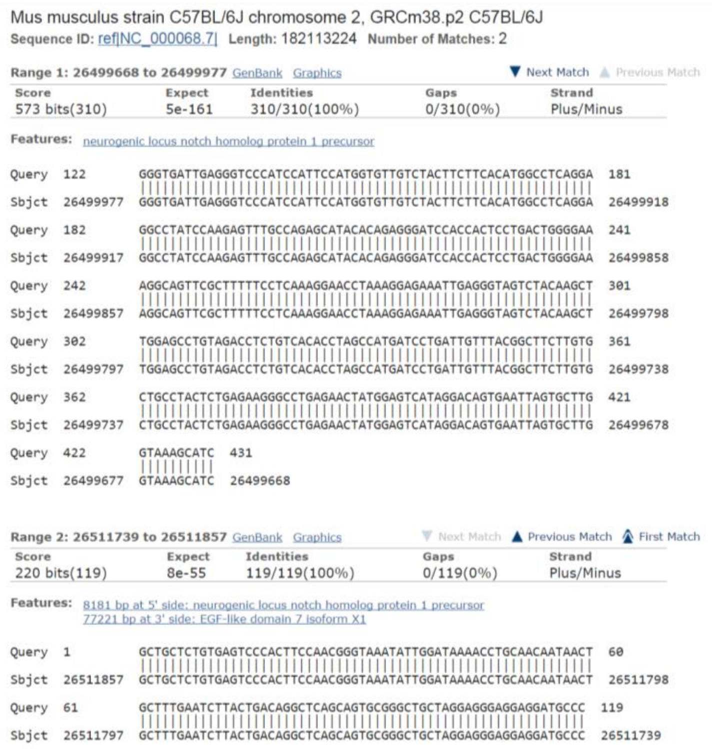

Above are shown sequencing data (forward and reverse) of Notch 5' Deletion PCR products for three TL samples. The left side shows identity with the area approximately 8 kb upstream of the start of *Notch1* (red box) and the right side shows identity with the internal *Notch1* sequence (blue box), with a variable region representing three unique re-joining events at the same breakpoint sites. A BLAST search of the PCR product confirms 11.8 Mb separating the two sequencing in the germline sequence.
