# Supplementary material for: Genetic Analysis of T Cell Lymphomas in Carbon Ion-Irradiated Mice Reveals Frequent Interstitial Chromosome Deletions: Implications for Second Cancer Induction in Normal Tissues during Carbon Ion Radiotherapy
Source: PLoS One. 2015 Jun 30;10(6):e0130666. doi: 10.1371/journal.pone.0130666 (PMC4488329; doi:10.1371/journal.pone.0130666)
Supplement: S1 Table — The crude incidence represents the number of mice diagnosed with TL at necropsy out of the total number of mice per group, without correction for competing risks. The body weight at the time of necropsy was significantly higher in mice receiving fractionated carbon irradiation compared to the same doses as a single exposure (P < 0.036, Bonferroni-Corrected Independent Samples T Test, two-tailed) an indication of the sparing effect on normal body development. (PDF) [file pone.0130666.s011.pdf]

S1 Table. TL Incidence and Characteristics of Tumours Included in the Study

Group	Crude TL Incidence (%)	№ Tumours Analysed (№ Male)	Body Weight at necropsy (g) *mean ± SEM*	Thymus Weight at necropsy (mg) *mean ± SEM*	Spleen Weight at necropsy (mg) *mean ± SEM*	Lifespan (days) *mean ± SD*	Lifespan (days) *min – max*
Gamma 1 × 4 Gy	16/98 (16.3)	16 (8)	26.5 ± 1.5	559 ± 58	386 ± 74	221 ± 77	106 – 378
Carbon 1 × 4 Gy	27/82 (32.9)	20 (11)	26.2 ± 1.0	557 ± 52	302 ± 47	195 ± 96	111 – 444
Carbon 1 × 4.8 Gy	34/88 (38.6)	28 (12)	24.4 ± 0.6	497 ± 44	296 ± 43	177 ± 79	88 – 514
Carbon 4 × 1 Gy	16/80 (20)	15 (7)	30.3 ± 1.1	486 ± 60	341 ± 45	222 ± 83	149 – 444
Carbon 4 × 1.2 Gy	43/80 (53.8)	38 (18)	27.7 ± 0.5	514 ± 37	423 ± 40	207 ± 66	140 – 445
