# Supplementary material for: Genetic Analysis of T Cell Lymphomas in Carbon Ion-Irradiated Mice Reveals Frequent Interstitial Chromosome Deletions: Implications for Second Cancer Induction in Normal Tissues during Carbon Ion Radiotherapy
Source: PLoS One. 2015 Jun 30;10(6):e0130666. doi: 10.1371/journal.pone.0130666 (PMC4488329; doi:10.1371/journal.pone.0130666)
Supplement: S2 Table — The threshold for low/high thymus weight (300 mg) represented the lowest quartile, and the threshold for high/low spleen weight (200 mg) seemed to separate a bimodal distribution likely indicating splenic infiltrating and non-infiltrating tumours. There was no significant association between the treatment group and the thymus/spleen organ weight status at necropsy (P = 0.40, Pearson’s Chi-squared Test), but there was a strongly significant negative correlation between thymus and spleen weight across the whole cohort (Pearson Correlation Coefficient = -0.33, P = 2.5×10^−4^) perhaps indicative of a tumour-specific preference for expansion in the thymus or the periphery. (PDF) [file pone.0130666.s012.pdf]

S2 Table. Distribution of Thymus and Spleen Mass for Tumours Included in the Study

Group	№ Tumours Analysed *(No. Male)*	Thymus > 300 mg Spleen > 200 mg	Thymus > 300 mg Spleen < 200 mg	Thymus < 300 mg Spleen > 200 mg	Thymus < 300 mg Spleen < 200 mg
		№ Tumours (% of Group Total)			
Gamma 1 × 4 Gy	16 (8)	8 (50%)	5 (31%)	3 (19%)	0 (0%)
Carbon 1 × 4 Gy	20 (11)	10 (50%)	9 (45%)	1 (5%)	0 (0%)
Carbon 1 × 4.8 Gy	28 (12)	13 (46%)	11 (39%)	3 (11%)	1 (4%)
Carbon 4 × 1 Gy	15 (7)	11 (73%)	3 (20%)	0 (0%)	1 (7%)
Carbon 4 × 1.2 Gy	38 (18)	21 (55%)	10 (26%)	7 (18%)	0 (0%)
