# Supplementary material for: Genetic Analysis of T Cell Lymphomas in Carbon Ion-Irradiated Mice Reveals Frequent Interstitial Chromosome Deletions: Implications for Second Cancer Induction in Normal Tissues during Carbon Ion Radiotherapy
Source: PLoS One. 2015 Jun 30;10(6):e0130666. doi: 10.1371/journal.pone.0130666 (PMC4488329; doi:10.1371/journal.pone.0130666)
Supplement: S3 Table — Here, 35 sequential carbon ion-induced TLs were examined at the time of necropsy for cell surface expression of the CD4, CD8, CD90 and CD45R cell surface antigens. All of the tumours were CD90+ and CD45R negative, indicative of T cell origin. The immunophenotypes included not only the standard CD4/CD8 double positive, double negative and single positive subtypes, but in addition, included tumours where one or both antigens were expressed along a continuum from low to high expression level (CD4+/- or CD8+/-). Three tumours also displayed two or more distinct immunophenotypes (as opposed to the aforementioned indistinct phenotypes). There was no significant association between treatment group and immunophenotype (P = 0.72, Pearson’s Chi-squared Test); however, the low numbers make it difficult to draw any firm conclusions. (PDF) [file pone.0130666.s013.pdf]

S3 Table. Distribution of Immunophenotypes between Carbon Ion Treatment Groups

Group	CD4+ CD8+	CD4- CD8-	CD4+ CD8-	CD4- CD8+	CD4+ CD8+/-	CD4+/- CD8+	CD4+/- CD8+-/	Mixed
	№ Tumours of Each Immunophenotype							
Carbon 1 × 4 Gy	5	-	2	-	-	-	1	-
Carbon 1 × 4.8 Gy	2	1	1	-	-	1	2	1
Carbon 4 × 1 Gy	1	-	-	-	-	2	1	-
Carbon 4 × 1.2 Gy	3	1	2	1	2	2	2	2
