# Supplementary material for: Genetic Analysis of T Cell Lymphomas in Carbon Ion-Irradiated Mice Reveals Frequent Interstitial Chromosome Deletions: Implications for Second Cancer Induction in Normal Tissues during Carbon Ion Radiotherapy
Source: PLoS One. 2015 Jun 30;10(6):e0130666. doi: 10.1371/journal.pone.0130666 (PMC4488329; doi:10.1371/journal.pone.0130666)
Supplement: S4 Table — (PDF) [file pone.0130666.s014.pdf]

S4 Table. Details of Sub-Cohort of TL for Array-based CGH Analysis

Tumour IDs	№ Tumours	Sex	Radiation	Total Dose	№ Fractions	Dose per Fraction	Age at First Irradiation
22717, 23362, 23500	3	Female	Carbon Ion	4.8 Gy	4	1.2 Gy	1 week
23364, 23291, 23741	3	Male	Carbon Ion	4.8 Gy	4	1.2 Gy	1 week
22490, 22708, 22570	3	Female	Carbon Ion	4 Gy	4	1.0 Gy	1 week
22705, 22544, 23476	3	Male	Carbon Ion	4 Gy	4	1.0 Gy	1 week
22767, 23584	2	Female	Carbon Ion	4.8 Gy	1	4.8 Gy	1 week
23721, 22578	2	Male	Carbon Ion	4.8 Gy	1	4.8 Gy	1 week
22533, 23265	2	Female	Carbon Ion	4 Gy	1	4 Gy	1 week
23473, 22803	2	Male	Carbon Ion	4 Gy	1	4 Gy	1 week
11478, 12118, 10691, 11498, 11534, 11697, 12077	7	Female	Gamma Ray	4 Gy	1	4 Gy	1 week
10976, 11360, 10910, 10989, 11463	5	Male	Gamma Ray	4 Gy	1	4 Gy	1 week
