# Supplementary material for: Genetic Analysis of T Cell Lymphomas in Carbon Ion-Irradiated Mice Reveals Frequent Interstitial Chromosome Deletions: Implications for Second Cancer Induction in Normal Tissues during Carbon Ion Radiotherapy
Source: PLoS One. 2015 Jun 30;10(6):e0130666. doi: 10.1371/journal.pone.0130666 (PMC4488329; doi:10.1371/journal.pone.0130666)
Supplement: S5 Table — All Notch1 PEST domain mutations are predicted to cause truncation by a direct nonsense mutation or a frameshift mutation with downstream premature stop codon. (PDF) [file pone.0130666.s015.pdf]

**S5 Table. Summary of *Notch1* PEST Domain Sequence Mutations**

| Mutation Class | Location [a] | Alteration | Predicted Protein Change | № Tumours (if >1) |
|---|---|---|---|---|
| Deletion | | | | |
| | +6951 | del 'G' | Frameshift | |
| | +7002-7027 | del 26 bp | Frameshift | |
| | +7053 | del 11 bp | Frameshift | |
| | +7080-7081 | del 'AC' | Frameshift | |
| | +7082 | GGCTGGCA>TTGCTGG' | Frameshift | |
| | +7082 | del CGG ins GC | Frameshift | |
| | +7273-7280 | del 'AGTCTGC' | Frameshift | |
| | +7454-7470 | del 17 bp | Frameshift | 2 |
| | +7455-7462 | del 'CCTCACCC' | Frameshift | |
| | +7469-7470 | del 2 bp | Frameshift | |
| | +7466-7566 | del 100 bp | Frameshift | |
| Point-Mutation | | | | |
| | +6913 | C>T | Nonsense: Q2305* | |
| | +7144 | C>T | Nonsense: Q2382* | |
| | +7171 | C>T | Nonsense: Q2391* | |
| | +7243 | C>T | Nonsense: Q2415* | |
| | +7429 | C>T | Nonsense: Q2477* | |
| | +7435 | C>T | Nonsense: Q2479* | |
| Insertion | | | | |
| | +6847 | G5>G6 | Frameshift | 4 |
| | +6926 | ins 'T' | Frameshift | |
| | +6984 | ins 'A' | Frameshift | |
| | +7020 | ins 'T' | Frameshift | |
| | +7052 | C4>C5 | Frameshift | |
| | +7052 | ‘C’>’GGTGT’ | Frameshift | |
| | +7056 | ins 'AACC' | Frameshift | |
| | +7080 | ins 'AGAC' | Frameshift | |
| | +7081 | ‘C'>'GA' | Frameshift | |
| | +7081 | ins 'C' | Frameshift | 2 |
| | +7081 | ins 'TTTTT' | Frameshift | |
| | +7081 | ins 'CCCC' | Frameshift | |
| | +7081 | ‘G'>'CC" | Frameshift | 2 |
| | +7081 | ‘G'>'CCCCCCTC' | Frameshift | |
| | +7082 | ‘G'>'CC' | Frameshift | |
| | +7082 | ins 'GAAA' | Frameshift | |
| | +7082 | ‘G'>'AA' | Frameshift | |
| | +7082 | ‘G'>'CC' | Frameshift | 2 |
| | +7082 | ‘G'>'CCCCC' | Frameshift | |
| | +7082 | ins 'CC' | Frameshift | |
| | +7082 | ins 'CCCC' | Frameshift | |
| | +7082 | ‘G'>'CCCTCCGC' | Frameshift | |
| | +7082 | ‘G'>'TT' | Frameshift | |
| | +7082 | ins 'CGCC' | Frameshift | |
| | +7083 | ins ‘CCCT’ | Frameshift | |
| | +7084 | ins 'G' | Frameshift | |
| | +7084 | ins 'GGGG' | Frameshift | |
| | +7087 | dup 29 bp | Frameshift | |
| | +7096 | dup 43 bp | Frameshift | |
| | +7183 | ins 'AGCGA' | Frameshift | |
| | +7185 | ins 'T' | Frameshift | |
| | +7193 | ins 8 bp | Frameshift | |
| | +7193 | ‘C'>'TT" | Frameshift | |
| | +7194 | ins 'CGCC' | Frameshift | |
| | +7194 | ins 'CCGC' | Frameshift | |
| | +7195 | ins 'GGGG' | Frameshift | |
| | +7196 | ins 'AAAG' | Frameshift | |
| | +7211 | ins 'CC' | Frameshift | |
| | +7216 | ins ‘GAG’ | Frameshift | |
| | +7228 | ins 'GGGA' | Frameshift | |
| | +7259 | C2>C4 | Frameshift | |
| | +7259 | ins ‘C’ | Frameshift | |
| | +7260 | ins 'CCCC' | Frameshift | |
| | +7260 | ins 'CCTC' | Frameshift | |
| | +7274 | ‘GTC'>'AGGGGTT' | Frameshift | |
| | +7294 | ins 'T' | Frameshift | |
| | +7343 | ‘T'>'GC' | Frameshift | |
| | +7377 | ins 'C' | Frameshift | |
| | +7406 | C4>C5 | Frameshift | |
| | +7407 | ins ‘A’ | Frameshift | |
| | +7423 | ins 'C' | Frameshift | |
| | +7446 | ins 'CACC' | Frameshift | |
| | +7460 | C5>C6 | Frameshift | |
| | +7463 | C4>C5 | Frameshift | |
| | +7464 | ins 'C' | Frameshift | |

[a] Location relative to ‘A’ of translation start ‘ATG’ codon.
