# Supplementary material for: Genetic Analysis of T Cell Lymphomas in Carbon Ion-Irradiated Mice Reveals Frequent Interstitial Chromosome Deletions: Implications for Second Cancer Induction in Normal Tissues during Carbon Ion Radiotherapy
Source: PLoS One. 2015 Jun 30;10(6):e0130666. doi: 10.1371/journal.pone.0130666 (PMC4488329; doi:10.1371/journal.pone.0130666)
Supplement: S6 Table — All Notch1 HD Domain mutations are predicted to produce in-frame codon deletions, insertions or substitutions. (PDF) [file pone.0130666.s016.pdf]

S6 Table. Summary of *Notch1* HD Domain Sequence Mutations

Mutation Class	Location [a]	Alteration	Predicted Protein Change	№Tumours (if >1)
**Deletion**				
	+4821-4829	del 9 bp	del R1608-A1610	
**Insertion**				
	+5142-5188	dup 48 bp	dup H1725-G1741	
	+4814	ins 'GGT'	V1605ins VV	
	+4999	ins 'CAACCT'	V1666ins VST	
**Point-Mutation**				
	+4721	T>C	L1574P	
	+5003	T>C	L1668P	11[b]
	+5009	T>G	I1670S	
	+5092	T>C	S1698P	
	+5096	T>C	L1699P	

[a] Location relative to 'A' of translation start 'ATG' codon.

[b] 8 tumours were C-TL, 3 were G-TL.
