# Supplementary material for: Genetic Analysis of T Cell Lymphomas in Carbon Ion-Irradiated Mice Reveals Frequent Interstitial Chromosome Deletions: Implications for Second Cancer Induction in Normal Tissues during Carbon Ion Radiotherapy
Source: PLoS One. 2015 Jun 30;10(6):e0130666. doi: 10.1371/journal.pone.0130666 (PMC4488329; doi:10.1371/journal.pone.0130666)
Supplement: S7 Table — (PDF) [file pone.0130666.s017.pdf]

S7 Table. Summary of *Ikzf1* Protein Coding Sequence Mutations

Mutation Class	Location [a]	Alteration	Predicted Protein Change	№Tumours (if>1)
**Aberrantly Spliced Transcripts**				
		del Exon 3-5	Aberrant protein	
		del Exon 3-6	Aberrant protein	
		del Exon 4	Aberrant protein	3
		del Exon 4-6	Aberrant protein	2
		del Exon 5	Aberrant protein	
		del Exon 5-6	Aberrant protein	
**Deletion**				
	+360	del 61 bp	Frameshift	
	+1053	del 'C'	Frameshift	
	+1253	'ACGC'>'CCT'	Frameshift	
	+1468	del 'AT'	Frameshift	
**Insertion**				
	Exon 1-2	del 17 bp from end of Exon 1, ins 150 bp upstream of Exon 2	Frameshift	
	Exon 2	'GT'>'TA' end of Exon 2, ins 74 bp downstream of Exon 2	Frameshift	
	Exon 5-6	ins 113 bp between Exon 5 and Exon 6 to form novel Exon	Frameshift	
	+56	C6>C7	Frameshift	2
	+141	C2>C7	Frameshift	
	+512	A2>A3	Frameshift	
	+764	ins 'C'	Frameshift	
	+810	ins 'G'	Frameshift	
	+825	ins 'CT'	Frameshift	
	+996	C6>C7	Frameshift	
	+1054	C6>C7	Frameshift	
	+1067	C6>C7	Frameshift	2
	+1140	G2>G9	Frameshift	
	+1232	ins 'GTGT'	Frameshift	
	+1286	'GC'>'GGG'	Frameshift	
**Point-Mutation**				
	+151	C>T	R51*	
	+431	C>T	P144L	
	+446	A>C	Q149P	
	+484	C>T	R162W	
	+485	G>C	R162P	
	+485	G>A	R162Q	
	+488	A>G	H163R	
	+488	A>C	H163P	
	+505	G>T	G169C	
	+518	T>C	F173C	
	+532	T>G	C178G	
	+539	A>G	Y180C	2
	+544	T>C	C182R	2
	+557	A>C	D186A	2
	+575	T>C	L192P	
	+589	G>A	R184Q	
	+637	C>T	R213*	
	+702	C>T	R222*	

[a] Location relative to 'A' of translation start 'ATG' codon.
