# Supplementary material for: Genetic Analysis of T Cell Lymphomas in Carbon Ion-Irradiated Mice Reveals Frequent Interstitial Chromosome Deletions: Implications for Second Cancer Induction in Normal Tissues during Carbon Ion Radiotherapy
Source: PLoS One. 2015 Jun 30;10(6):e0130666. doi: 10.1371/journal.pone.0130666 (PMC4488329; doi:10.1371/journal.pone.0130666)
Supplement: S8 Table — All Trp53 mutations were found in carbon ion-induced tumours. (PDF) [file pone.0130666.s018.pdf]

S8 Table. Summary of *Trp53* Protein Coding Sequence Mutations

Mutation Class	Location [a]	Alteration	Predicted Protein Change	№Tumours (if >1)
**Deletion**				
	Exon 2 – 4	del Exon 2 – 4	Aberrant protein	
**Insertion**				
	+726	ins ‘GC’	Frameshift	
**Point-Mutation**				
	+329	T>G	F110C	
	+404	C>T	A135V	3
	+508	G>A	V170M	
	+578	G>C	R193P	
	+628	C>T	R210C	
	+632	A>G	H211R	
	+638	T>G	V213G	
	+692	A>G	Y231C	
	+722	G>A	G241E	
	+801	T>G	F267L	
	+808	C>T	R270C	

[a] Location relative to ‘A’ of translation start ‘ATG’ codon.
