# Supplementary material for: Genetic Analysis of T Cell Lymphomas in Carbon Ion-Irradiated Mice Reveals Frequent Interstitial Chromosome Deletions: Implications for Second Cancer Induction in Normal Tissues during Carbon Ion Radiotherapy
Source: PLoS One. 2015 Jun 30;10(6):e0130666. doi: 10.1371/journal.pone.0130666 (PMC4488329; doi:10.1371/journal.pone.0130666)
Supplement: S9 Table — (PDF) [file pone.0130666.s019.pdf]

S9 Table. Summary of *Pten* Protein Coding Sequence Mutations

Mutation Class	Location [a]	Alteration	Predicted Protein Change	№Tumours (if>1)
**Aberrantly Spliced Transcripts**				
		del Exon 2	Aberrant protein	2
		del Exon 2-8	Aberrant protein	
		del Exon 2-9	Aberrant protein	
		del Exon 3-5	Aberrant protein	2
		del Exon 4	Aberrant protein	2
		del Exon 4 & 5	Aberrant protein	
		del Exon 5	Aberrant protein	2
		del Exon 7	Aberrant protein	2
		del Exon 7-8	Aberrant protein	
		del Exon 8	Aberrant protein	2
**Deletion**				
	+261-338	del 77 bp	Frameshift	
	+968	del AA ins T	Frameshift	
	+968	del ‘A’	Frameshift	
	+332-491	del 161 bp	Frameshift	
**Insertion**				
	+13	ins ‘T’	Frameshift	
	+106	ins 'T'	Frameshift	
	+170	T5>T6	Frameshift	
	+312	ins 'CTTC'	Frameshift	
	+546	A6>A7	Frameshift	4
	+720	‘T’>‘TTATGG’	Frameshift	
	+938	A2>A3	Frameshift	
	+963	A6>A7	Frameshift	3
	+988	ins 'GGGTG'	Frameshift	
	+989	ins 'C'	Frameshift	
	+1029	dup (8 bp)	Frameshift	
**Point-Mutation**				
	+95	T>G	I32S	
	+146	A>T	N49I	
	+277	C>T	H93Y	
	+372	T>G	C124W	
	+389	G>A	R130Q	
	+391	A>C	T131P	
	+398	T>G	V133G	
	+419	T>A	L140*	
	+476	G>C	R159T	
	+512	A>G	Q171R	
	+517	C>T	R173C	
	+542	T>C	L181P	
	+796	A>T	K266*	
	+830	C>T	T277M	
	+974	T>G	L325R	
	+1003	C>T	R335*	

[a] Location relative to ‘A’ of translation start ‘ATG’ codon.
